# Comparative transcriptome analysis reveals important roles of nonadditive genes in maize hybrid An’nong 591 under heat stress

**DOI:** 10.1186/s12870-019-1878-8

**Published:** 2019-06-24

**Authors:** Yang Zhao, Fangxiu Hu, Xingen Zhang, Qiye Wei, Jinlei Dong, Chen Bo, Beijiu Cheng, Qing Ma

**Affiliations:** 1 The National Engineering Laboratory of Crop Stress Resistance Breeding, School of Life Sciences, Anhui Agricultural University, Hefei, China; 2 Key Laboratory of Crop Biology of Anhui Province, School of Life Sciences, Anhui Agricultural University, Hefei, China

**Keywords:** Maize, RNA-Seq, Expression pattern, Heterosis, Heat stress

## Abstract

**Background:**

Heterosis is the superior performance of F_1_ hybrids relative to their parental lines for a wide range of traits. In this study, expression profiling and heterosis associated genes were analyzed by RNA sequencing (RNA-Seq) in seedlings of the maize hybrid An’nong 591 and its parental lines under control and heat stress conditions.

**Results:**

Through performing nine pairwise comparisons, the maximum number of differentially expressed genes (DEGs) was detected between the two parental lines, and the minimum number was identified between the F_1_ hybrid and the paternal lines under both conditions, which suggested greater genetic contribution of the paternal line to heat stress tolerance. Gene Ontology (GO) enrichment analysis of the 4518 common DEGs indicated that GO terms associated with diverse stress responses and photosynthesis were highly overrepresented in the 76 significant terms of the biological process category. A total of 3970 and 7653 genes exhibited nonadditive expression under control and heat stress, respectively. Among these genes, 2253 (56.8%) genes overlapped, suggesting that nonadditive genes tend to be conserved in expression. In addition, 5400 nonadditive genes were found to be specific for heat stress condition, and further GO analysis indicated that terms associated with stress responses were significantly overrepresented, and 60 genes were assigned to the GO term response to heat. Pathway enrichment analysis indicated that 113 genes were involved in spliceosome metabolic pathways. Nineteen of the 33 overlapping genes assigned to the GO term response to heat showed significantly higher number of alternative splicing (AS) events under heat stress than under control conditions, suggesting that AS of these genes play an important role in response to heat stress.

**Conclusions:**

This study reveals the transcriptomic divergence of the maize F_1_ hybrid and its parental lines under control and heat stress conditions, and provides insight into the underlying molecular mechanisms of heterosis and the response to heat stress in maize.

**Electronic supplementary material:**

The online version of this article (10.1186/s12870-019-1878-8) contains supplementary material, which is available to authorized users.

## Background

Heterosis, or hybrid vigor, refers to the superior agronomic performance of heterozygous F_1_ plants compared with that of their homozygous parents [1, 2]. Although this phenomenon has been widely exploited by plant breeders for decades, the underlying genetic and molecular mechanisms of heterosis are not yet completely understood [3]. Before formulation of molecular genetics concepts, the classical quantitative genetic explanations for heterosis focused mainly on two models, the dominance (or complementation) hypothesis and the overdominance hypothesis. The first hypothesis assumes that the favorable alleles associated with heterosis from either parent are dominant at different loci that are complemented and can thereby mask deleterious alleles in the F_1_ hybrid [4, 5]. The overdominance hypothesis states that heterosis is a consequence of favorable interactions of alleles at heterozygous loci that are superior to the effect of any two homozygous alleles [1, 6]. In addition, epistasis has been demonstrated to contribute to heterosis, which refers to the intergenic interaction between two or more favorable genes of the parents [2, 7, 8]. Evidence for each hypothesis has been presented [3, 9–13]; however, there is still no consensus on the mechanism for heterosis.

Increasing evidence indicates that differential gene expression in parental lines and hybrids may be responsible for heterosis [3, 7, 14, 15]. With the development of transcript profiling technology, differentially expressed genes (DEGs) between a hybrid and its parents can be identified and used to explore the possible molecular mechanisms of heterosis. For example, a total of 829 and 4186 DEGs were identified by RNA sequencing (RNA-Seq) between a rice hybrid and its parents at the tillering and heading stages, respectively [16]. Transcriptome analysis of the primary root of the maize inbred lines B73 and Mo17 and their reciprocal hybrids revealed that 42–57% of expressed genes were differentially expressed between one of the parents and one of the hybrids, and about 12% of expressed genes were detected as nonadditive genes in both hybrids [15]. Microarray analyses for expression profiling in seedlings of the same maize genotypes (B73, Mo17 and Mo17 × B73) showed that 22% of the differentially regulated expressed sequence tags (ESTs) exhibited nonadditive expression [3]. Studies of heterosis-associated genes for a number of traits in maize, rice, *Medicago*, and *Arabidopsis*, have also been performed based on expression profiling [17–21]. Gene expression patterns can be divided into either additive or nonadditive expression on the basis of differential expression in the hybrids compared with that of the parents. Nonadditive genes, which refers to genes in the hybrids that show significantly different expression from the average of their parents (the mid-parent value), have been suggested to be associated with heterosis. Conversely, additive expression refers to genes for which a hybrid accumulates a level of transcripts equal to the mid-parent value. Nonadditive gene expression in hybrids includes levels of transcripts equal to the high or low parent (high or low parent dominance), above the high parent (overdominance), or below the low parent (underdominance) [3, 15, 21–25]. For example, using transcriptome analysis of the mature embryo, a total of 4766 and 4081 transcripts were identified as DEGs between the maize hybrid Zhengdan 958 and its parental lines (Chang 7–2 and Zheng 58, respectively) and were further divided into additive, paternal dominance, maternal dominance, overdominance, and underdominance in accordance with the different expression patterns [26].

Heterosis confers superior performance for a wide range of traits such as increased biomass, development rate, and grain yield, but also tolerance to environmental stresses [27]. High temperature is one of the most serious abiotic stresses that affect plant growth and development, including important traits such as pollen fertility and photosynthate supply [28, 29]. Global climatic changes have caused severe crop yield losses, and it is predicted that the increase in global surface temperature will exceed 2 °C by the end of this century [30]. In plants, transcriptome analyses have indicated that transcription of thousands of genes altered in response to heat stress [31–33]. However, limited information is available on the underlying heterosis-associated genes in response to heat stress. Transcriptome analysis has been used to identify DEGs of the maize inbred lines B73 and Mo17 and their reciprocal F_1_ hybrids in response to drought, and revealed that 9230 (35%) and 7185 (27%) of expressed genes exhibited nonadditive expression under control and water deficit stress in the Mo17 × B73 hybrid, respectively. Importantly, 47% of the nonadditively expressed genes overlapped between the two treatments, which suggested important roles for these genes in response to drought [23]. Therefore, identification of heterosis-associated genes is crucial to reveal candidate genes with important functions in the response to heat stress and the underlying mechanism of heterosis.

Maize is one of the most important cereal crops worldwide. In recent years, high temperature has become a serious environmental stress affecting maize production. For example, extremely high temperatures (~ 40 °C) during the flowering period resulted in severe yield losses in Huang-Huai-Hai plain of China in 2016–2018. Planting heat stress-tolerant cultivars is one of the most effective approaches to prevent stress damage. The maize hybrid An’nong 591, which is highly resistant to high temperatures, is suitable for planting in the Huang-Huai-Hai region. To investigate the molecular mechanisms in the response to heat stress, the expression profiles of An’nong 591 and its parental lines were compared in seedlings under control and 42 °C treatments by RNA-Seq, and heterosis-associated genes were analyzed to explore the underlying mechanism of heterosis. The results lay an important foundation for understanding heat-tolerance mechanisms in maize hybrids, and provide insight into the underlying molecular basis of heterosis.

## Results

### Characterization of maize hybrid An’nong 591 and its parental lines

**Fig. 1:**
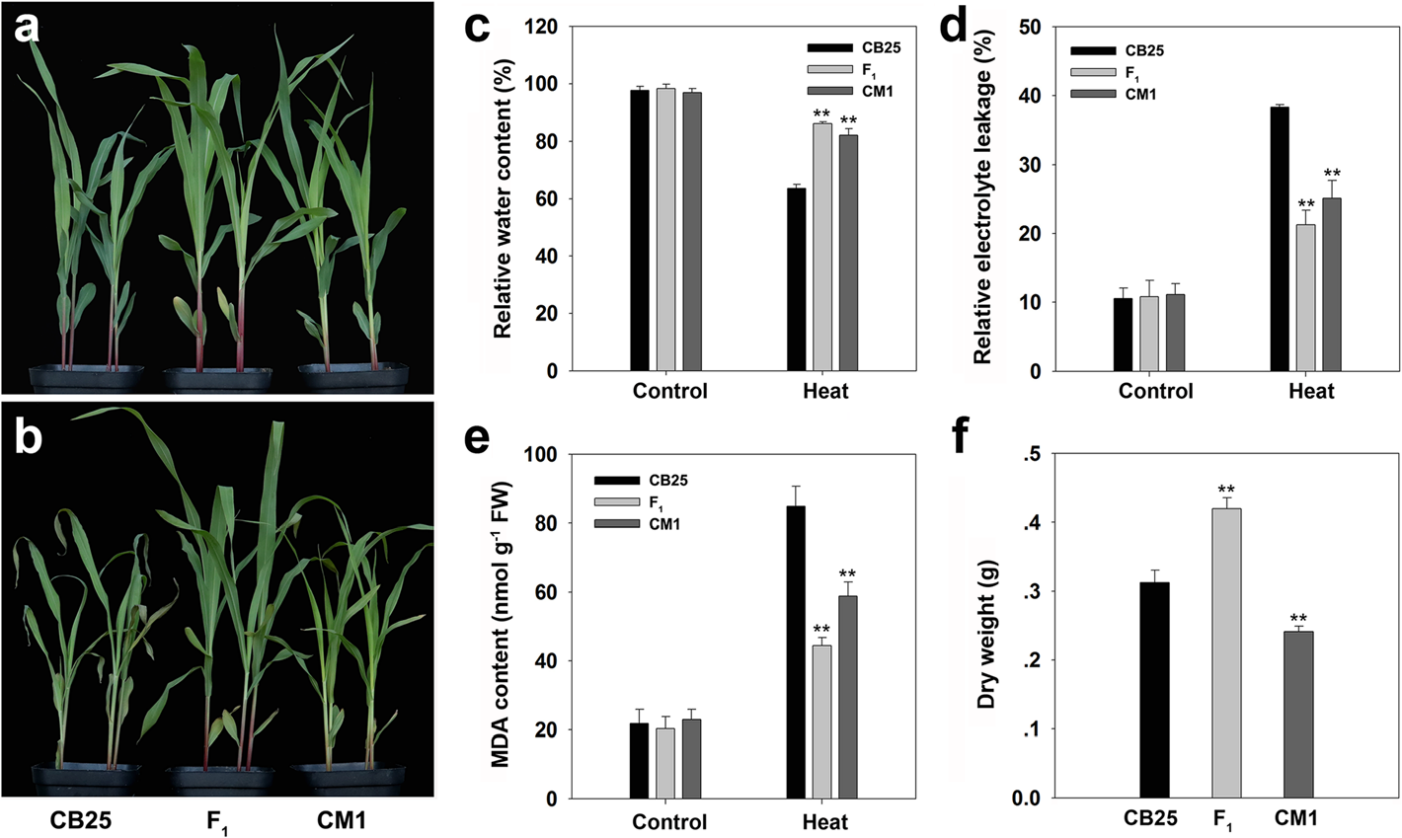
Phenotypic characteristics of the F_1_ hybrid An’nong 591 and its parental lines. **a** and **b** Phenotypes of the F_1_ hybrid and its parental lines in response to heat stress for 2 d. **c**, **d** and **e** Relative water content, relative electrolyte leakage, and malondialdehyde content in the F_1_ hybrid and its parental lines under control and heat treatment conditions. **f** Dry weight of 15 individual seedlings per genotype at 14 d after planting. Data represent mean values ± SD. ** indicates significant differences compared with the maternal line CB25 under the same conditions (*P* < 0.01)

Seedlings of the maize hybrid An’nong 591, its maternal line (CB25), and paternal line (CM1) were used to determine their phenotypic characters. When the third leaf was fully expanded, the seedlings of each genotype were treated with 42 °C/35 °C (day/night temperature) for two days to evaluate their phenotypic response to high temperature. Seedlings of CB25 showed severe leaf rolling and wilting in contrast with An’nong 591 and CM1 (Fig. 1a and b). CB25 also exhibited significantly lower RWC after heat stress compared with that of An’nong 591 or CM1 (Fig. 1c), whereas no significant difference was observed among the three genotypes under control growth conditions. CB25 exhibited significantly higher REL and MDA content after treatment compared with those of hybrid An’nong 591 and CM1 (Fig. 1d and e). These results indicated that An’nong 591 and CM1 are more tolerant to heat stress than CB25. Under control growth conditions, the dry weight of An’nong 591 was significantly higher than that of its parental lines (Fig. 1f). We calculated the MPH and HPH values and found significant MPH (51.42%) and HPH (34.21%) for the dry weight of An’nong 591 (*P* < 0.01).

### RNA-Seq and mapping reads to the maize genome

**Fig. 2:**
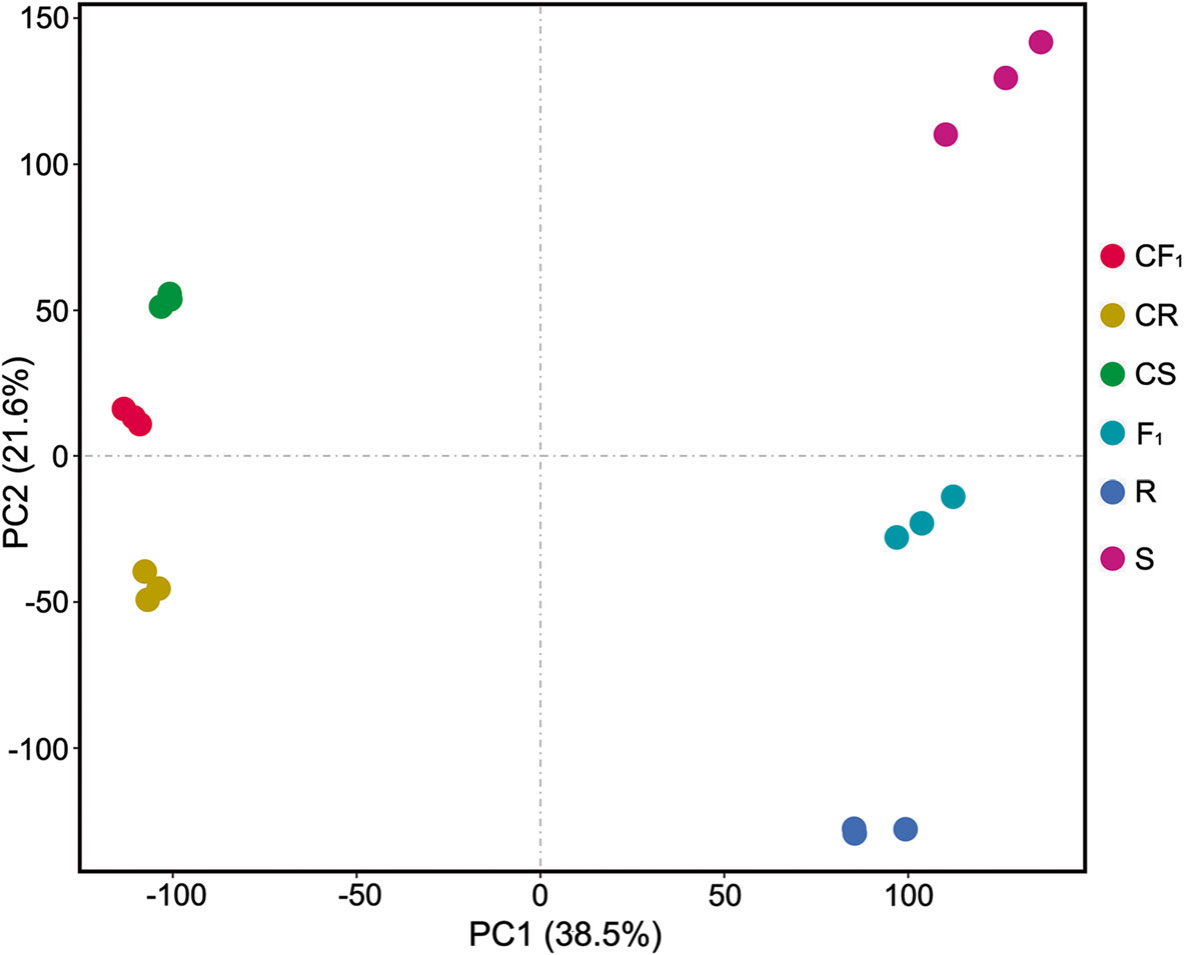
Principal component analysis of the relationship among samples of the three genotypes. The percentage of variation among samples explained by each principal component is shown on the *x*-axis and *y*-axis, respectively. CS, CR, and CF_1_ represent maternal line CB25, paternal line CM1, and F_1_ hybrid An’nong 591 under control conditions, respectively; S, R and F_1_ represent the corresponding genotypes under heat treatment

To identify genes that were responsive to heat stress in the seedlings, we used transcriptome sequencing to investigate global gene expression. The RNA-Seq analysis yielded 39.45–49.88 million raw reads per cDNA library with an average read length of 150 bp. After removing the reads containing adapter or poly (N) containing and the low quality sequences, on average, 45.81 million clean reads (6.82 Gb clean data) were obtained for each replicate. Among the 18 sequencing libraries, 72.57–75.65% of the clean reads were uniquely mapped to the maize reference genome (ZmB73_RefGen_v3). The percentages of phred scores at the Q30 level (error probability less than 0.1%) ranged from 92.36 to 93.26%, and the GC content ranged from 54.04 to 58.74%. Detailed information on the RNA-Seq data is listed in Additional file 8 Table S2. The normalized FPKM was used to determine the gene expression level for each sample. Genes with an average FPKM ≥1 in at least one sample of the three genotypes was considered to be expressed. As a result, 22,195 genes (56.2%) of the 39,475 high confidence gene models of the AGPv3 were expressed in at least one sample. Compared with gene expression under normal conditions, the overall abundance of genes was usually higher after heat stress (Additional file 1 Figure S1). Principal component analysis (PCA) was performed to examine the relationships among samples of the three genotypes under the control and heat-stress conditions. The first principal component (PC1) accounted for 38.5% of the variance, whereas the second principal component (PC2) accounted for 21.6% of the variance. The three biological replicates of each sample clustered closely together, which supported the high transcriptomic correlation (Fig. 2). The identity of the biological replicates was also verified using Pearson’s correlation analysis. The hierarchical clustering indicated that most of the correlation coefficients (*R*^2^) between the biological replicates were greater than 0.95 (Additional file 2 Figure S2), whereas only one correlation coefficient (between S1 and S3) was close to 0.80. Overall, these results indicated high reproducibility of the biological replicates based on RNA-Seq.

### Identification of DEGs in response to heat stress

Significantly DEGs were screened between the different samples with the criteria of fold change ≥2 and FDR ≤ 5%. To determine the genes that were differentially expressed in the three genotypes, nine pairwise comparisons (CS vs. CF_1_, CR vs. CF_1_, CS vs. CR, S vs. F_1_, R vs. F_1_, S vs. R, CF1 vs. F_1_, CR vs. R, and CS vs. S) were performed. Hierarchical cluster analysis indicated that the three biological replicates of each sample were clustered in close proximity to each other, which further reflected the highly reproducible results of RNA-Seq (Additional file 3 Figure S3). Under control conditions, a total of 2449 (1620 up- and 829 down-regulated), 2150 (1418 up- and 732 down-regulated) and 5789 (2952 up- and 2837 down-regulated) DEGs were identified for the comparisons CS vs. CF_1_, CR vs. CF_1_, and CS vs. CR, respectively. We identified 638 DEGs that were in common among these three pairs (Additional file 4 Figure S4a and S4d). After heat stress, the number of DEGs was much higher than that observed under control conditions. A total of 4721 (2521 up- and 2200 down-regulated), 3011 (1907 up- and 1104 down-regulated) and 8468 (4156 up- and 4312 down-regulated) DEGs were identified for the comparisons S vs. F_1_, R vs. F_1_, and S vs. R, respectively. We identified 1309 DEGs that were in common among these three pairs (Additional file 4 Figure S4b and S4e). A total of 9351 (5058 up- and 4293 down-regulated), 8055 (4377 up- and 3678 down-regulated), and 9624 (5108 up- and 4516 down-regulated) DEGs were identified for the pairs of CF_1_ vs. F_1_, CR vs. R and CS vs. S, respectively, to determine possible DEGs involved in the response to heat stress, of which 4629 DEGs were identified that were in common among these three pairs (Additional file 4 Figure S4c and S4f).

### Functional classification of common DEGs

**Fig. 3:**
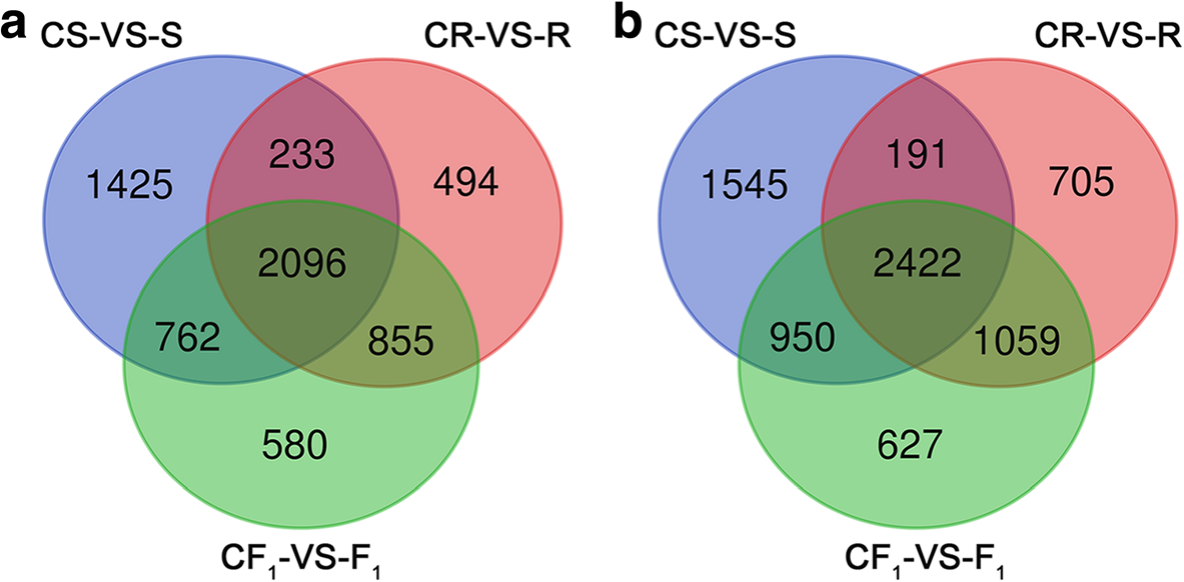
Venn diagram of common differentially expressed genes between the control and heat treatment. **a** Total number of common down-regulated DEGs. **b** Total number of common up-regulated DEGs

**Fig. 4:**
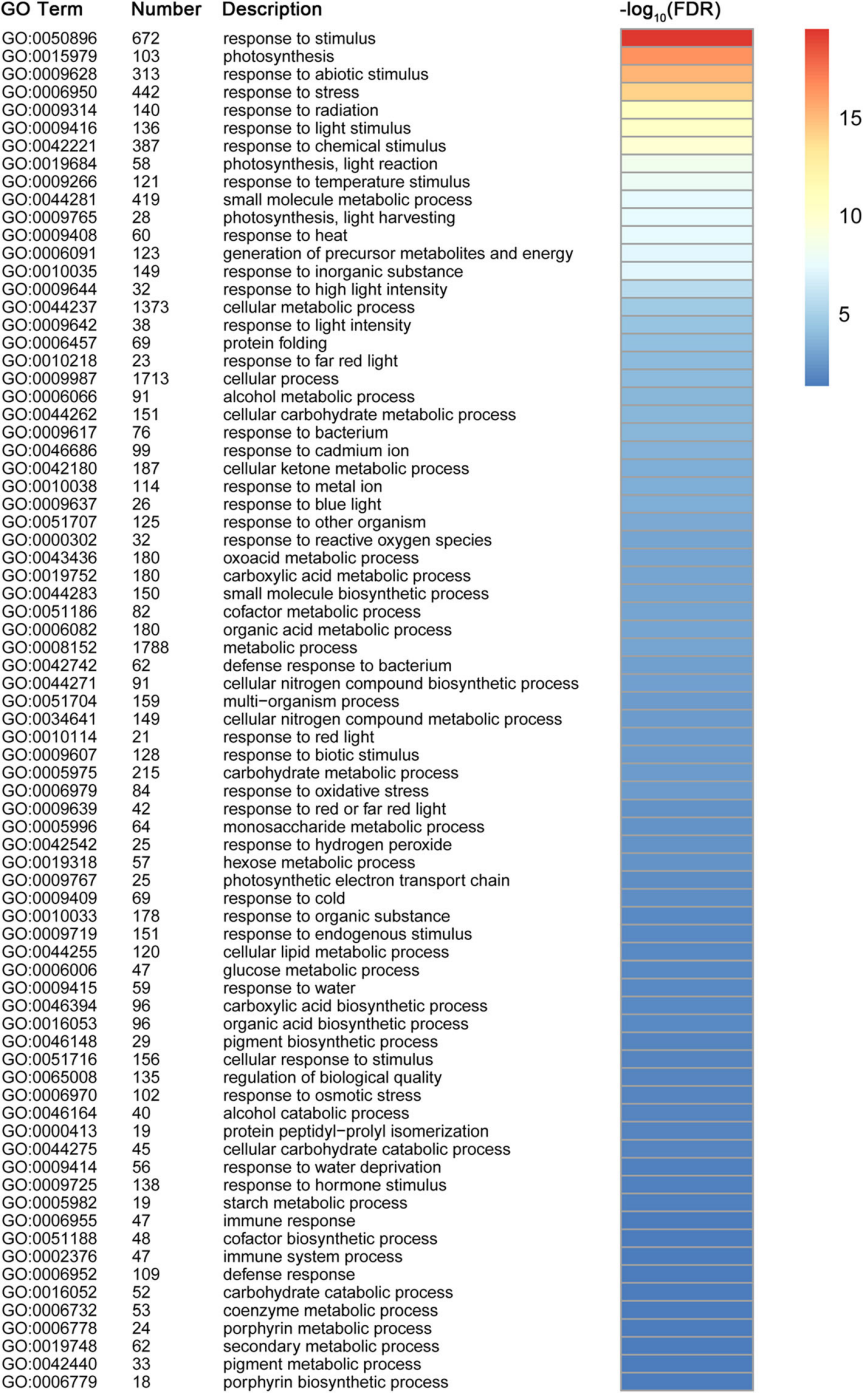
Enrichment of GO biological process terms for the 4518 common differentially expressed genes. GO enrichment analysis was performed using the agriGO analysis tool. Different colors in the right represent the significance levels. Only GO terms with significant levels of enrichment (FDR ≤ 5%) are shown

Among the 4629 DEGs in common among the control versus heat treatment samples, 2096 common down-regulated and 2422 common up-regulated genes were identified (Fig. 3). GO functional classification was performed using the web-based agriGO software to determine the biological processes in which the common DEGs are involved. Seventy-six GO biological process terms were significantly enriched (FDR ≤ 5%) among the 4518 common DEGs identified (Fig. 4 and Additional file 5 Figure S5). Many enriched GO terms were associated with stress and light responses, including response to stimulus (GO:0050896), response to abiotic stimulus (GO:0009628), response to stress (GO:0006950), response to photosynthesis (GO:0015979), and response to light stimulus (GO:0009416). Importantly, 60 genes were identified in the enriched GO term response to heat (GO:0009408), and 121 genes in the enriched GO term response to temperature stimulus (GO:0009266). These results suggested these genes perform important roles in response to heat stress.

GO enrichment analyses were also performed separately for the common down and up-regulated genes to investigate the differences in heat response mechanism. The results revealed a significant difference between down- and up-regulated DEGs in biological processes under different GO categories. A total of 119 GO biological process terms were significantly enriched for the common down-regulated DEGs. The GO terms associated with photosynthesis (GO:0015979), photosynthesis, light reaction (GO:0019684), generation of precursor metabolites and energy (GO:0006091), photosynthesis, light harvesting (GO:0009765), response to stimulus (GO:0050896), response to light stimulus (GO:0009416), response to radiation (GO:0009314), and response to abiotic stimulus (GO:0009628) were overrepresented in all categories (Additional file 9 Table S3). With regard to the up-regulated DEGs, the GO terms response to heat (GO:0009408), protein folding (GO:0006457), response to stress (GO:0006950), response to temperature stimulus (GO:0009266), and response to stimulus (GO:0050896), were overrepresented among the 19 significant enriched terms. In particular, we observed considerable enrichment of DEGs in the GO terms response to heat (53 genes) with highly significant FDR values (Additional file 10 Table S4).

**Fig. 5:**
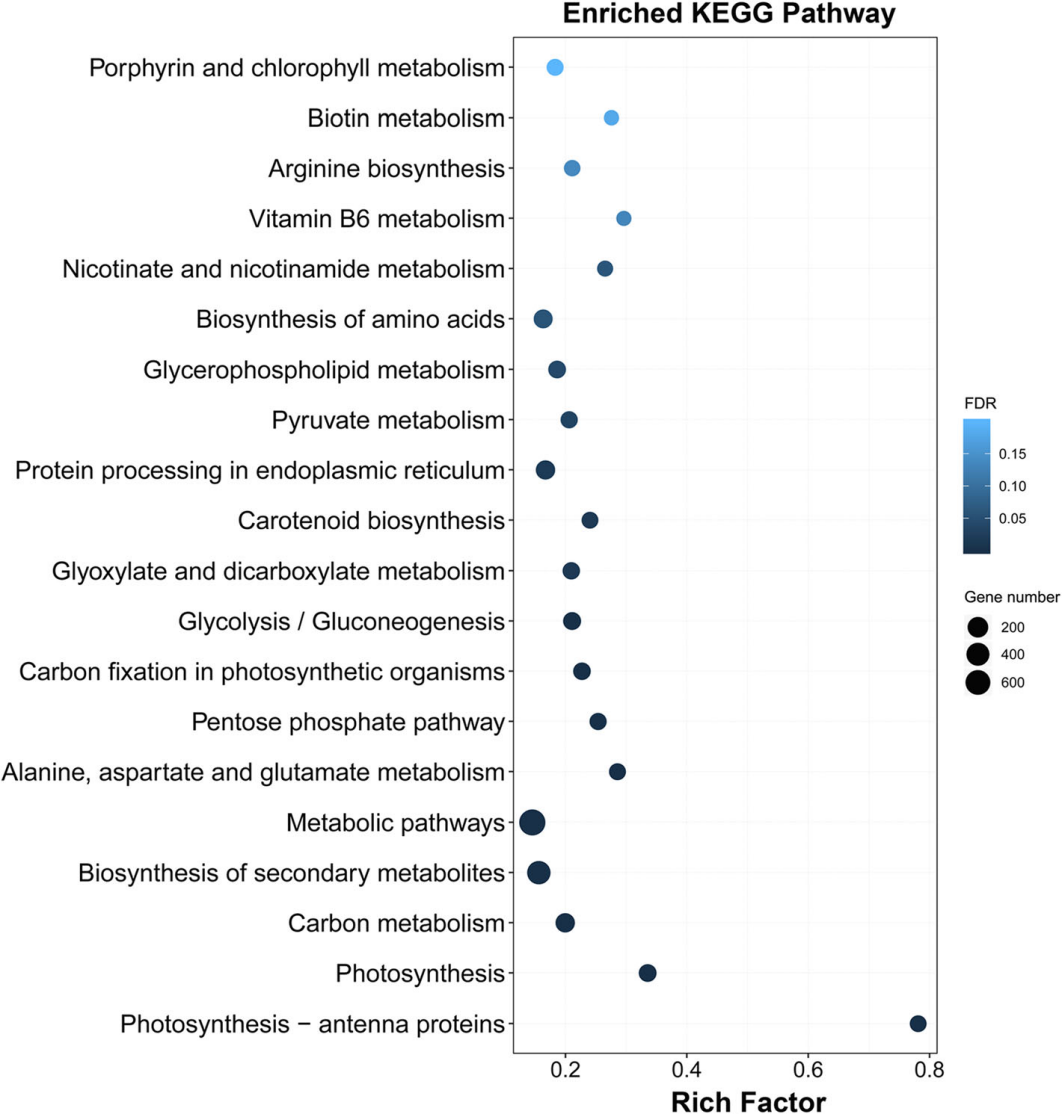
KEGG enrichment analysis of the 4518 common differentially expressed genes. The size of the dot indicates the number of DEGs involved in the pathway. The color scale indicates the significance level (FDR). The rich factor is the ratio between the number of DEGs and all genes enriched in the pathway

KEGG pathway analysis was further performed for these common DEGs. A total of 14 significant KEGG pathways (FDR ≤ 5%) were enriched for the 4518 DEGs (Fig. 5), including photosynthesis-antenna proteins, photosynthesis, carbon metabolism, biosynthesis of secondary metabolites, metabolic pathways, alanine, aspartate and glutamate metabolism, pentose phosphate pathway, carbon fixation in photosynthetic organisms, glycolysis/gluconeogenesis, glyoxylate and dicarboxylate metabolism, carotenoid biosynthesis, protein processing in endoplasmic reticulum, pyruvate metabolism, and glycerophospholipid metabolism. Among these pathways, the pathway category with the maximum number of genes was metabolic pathways with 772 genes.

### Clustering analysis and verification of RNA-Seq data by qRT-PCR

**Fig. 6:**
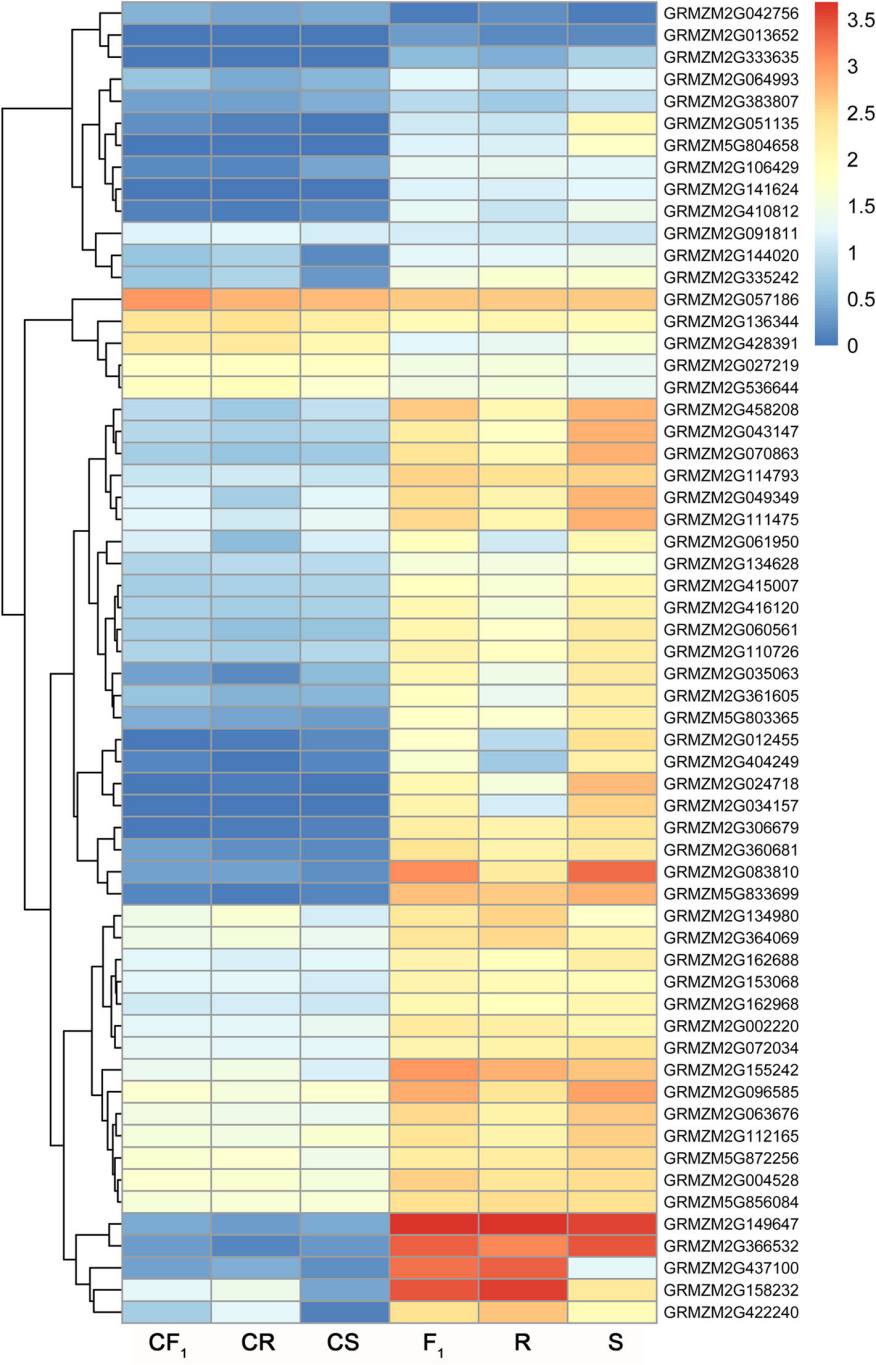
Hierarchical clustering of the 60 differentially expressed genes enriched in the GO term response to heat. The heatmap was drawn with the R package pheatmap. The color scale at the right of the figure indicates the gene expression levels transformed by log_10_ (FPKM + 1)

**Fig. 7:**
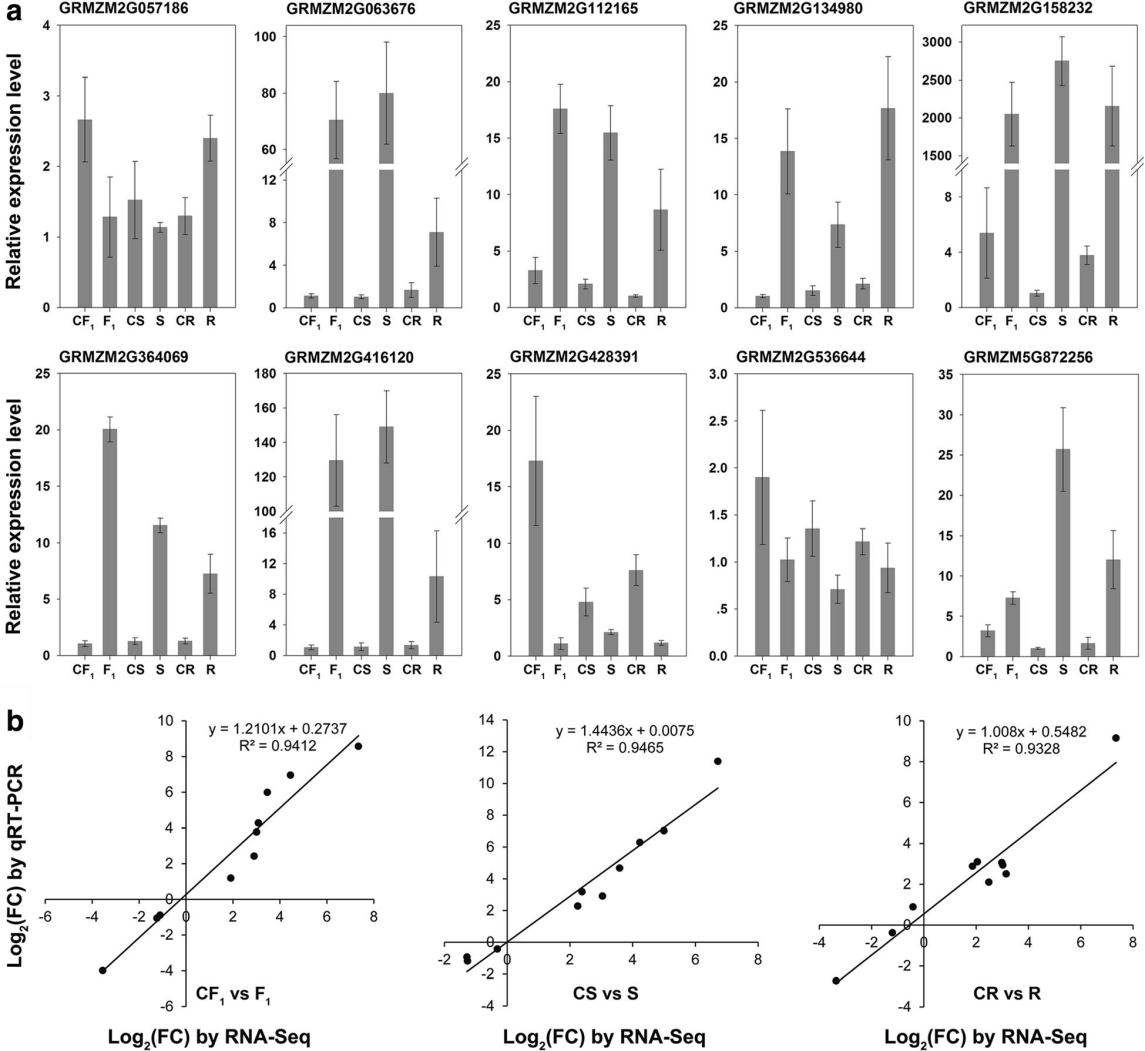
Quantitative RT-PCR validation of differentially expressed genes characterized by RNA sequencing. **a** Relative expression level of 10 randomly selected genes. **b** Correlation analysis of the RNA-Seq data (log_2_ FC) and qRT-PCR (log_2_ FC) for the F_1_ hybrid (CF_1_ vs. F_1_), maternal line (CS vs. S), and paternal line (CR vs. R) under control and heat stress conditions, respectively

According to the GO enrichment analysis of the 4518 common DEGs, we performed cluster analysis of the 60 genes identified for the enriched GO term response to heat (GO:0009408) using the R package pheatmap. The average expression levels of these genes were transformed by log_10_ (FPKM + 1) using three biological replicates of each sample. The majority of the 60 genes showed a low expression level under control conditions, whereas significantly up-regulated expression was detected after heat treatment (Fig. 6), suggesting that these genes perform important roles in response to heat stress. Ten genes among the 60 DEGs were randomly selected and validated by qRT-PCR using the same RNA samples as used for the RNA-Seq library construction. The ratio of the relative expression level between control and heat-stressed samples was transformed by log_2_ of the fold change detected by qRT-PCR, and used to compare with the results of RNA-Seq data. The qRT-PCR data showed a significant correlation (*R*^2^ = 0.9328–0.9465) with the RNA-Seq data for each of the three genotypes (Fig. 7), which supported the reliability of expression patterns revealed by RNA-Seq.

### Expression patterns of gene action in An’nong 591 and its parental lines

**Fig. 8:**
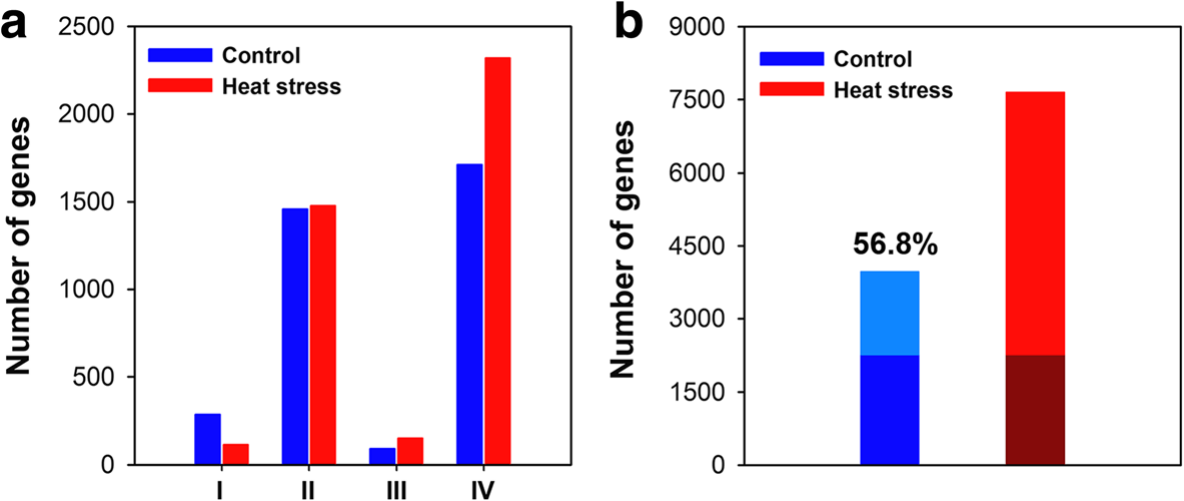
Expression pattern of gene action and nonadditive genes between F_1_ hybrid and its parental lines. **a** Expression patterns of gene action between the F_1_ hybrid and its parental lines under the control and heat stress conditions. **b** Overlap of nonadditive genes under control and heat stress conditions. The percentages of dark color indicate overlapping nonadditive genes

According to the differences in expression between hybrids and their parental lines, genes can be classified into five expression patterns, namely co-silence expression of the parental lines (Type I: genes are expressed in both of the parental lines, but not in F_1_ hybrid), parental-specific expression (Type II: genes are expressed only in one of the parental lines), hybrid-specific expression (Type III: genes are expressed only in F_1_ hybrid), single-parental consistent expression (Type IV: genes are expressed in F_1_ and one of the parental lines), and co-expression of the hybrid and parental lines (Type V) [26]. In the present study, under the control conditions, a total of 288, 1459, 93, and 1713 genes were identified for Types I to IV, respectively, whereas 116, 1478, 153, and 2321 genes were detected for the four groups under heat treatment (Fig. 8a). For Types II to IV, the number of genes detected in response to heat stress was larger than that under the control conditions.

Previous studies have indicated that Types I to IV are associated with qualitative differences in gene expression, meanings presence/absence variation of these genes. Type V is associated with quantitative differences in gene expression, and the difference in expression patterns between hybrids and their parental lines is predominantly associated with this type [3, 26]. A total of 12 classes of DEGs were defined according to the expression pattern in the hybrid relative to its parental lines in the present study (Table 1). A total of 6520 and 11,697 genes were identified in the 12 classes under the control and heat stress conditions, respectively. The maximum number of genes was observed in classes 1–6 and 13–18, in which the expression level in An’nong 591 was intermediate between that of its parents CB25 and CM1 or close to one of the parental inbred lines. Under control conditions, 137 genes showed underdominant expression (classes 7–9), whereas 183 genes showed overdominant expression (classes 10–12). Under heat stress, 354 genes (classes 19–21) and 463 genes (classes 22–24) genes in the hybrid displayed underdominant and overdominant expression, respectively. Among the 6520 genes identified under control condition, 2326 genes displayed nonadditive expression, accounting for 10.50% of the total number of expressed genes (22,195), whereas more than twice as many genes (5817, 26.2%) showed nonadditive expression under heat stress.

**Table 1:** Identification of nonadditive genes under control and heat stress conditions

Class	Expression Patterns	Total No. of Genes	No. of Nonadditive Genes
1	CR > CF_1_ > CS	651	122
2	CS > CF_1_ > CR	915	188
3	CR = CF_1_ < CS	801	358
4	CR = CF_1_ > CS	1702	511
5	CR > CF_1_ = CS	772	418
6	CR < CF_1_ = CS	1359	409
7	CR > CS > CF_1_	17	17
8	CS > CR > CF_1_	6	6
9	CR = CS > CF_1_	114	114
10	CF_1_ > CR > CS	12	12
11	CF_1_ > CS > CR	16	16
12	CF_1_ > CS=CR	155	155
	Others	15,675	1644
	Total	22,195	3970
13	R > F_1_ > S	1962	613
14	S > F_1_ > R	1838	850
15	R = F_1_ < S	2088	1398
16	R = F_1_ > S	2068	883
17	R > F_1_ = S	1254	571
18	R < F_1_ = S	1670	685
19	R > S > F_1_	12	12
20	S > R > F_1_	91	91
21	R = S > F_1_	251	251
22	F_1_ > R > S	86	86
23	F_1_ > S > R	40	40
24	F_1_ > S = R	337	337
	Others	10,498	1836
	Total	22,195	7653

### Comparative analysis of nonadditive genes

**Fig. 9:**
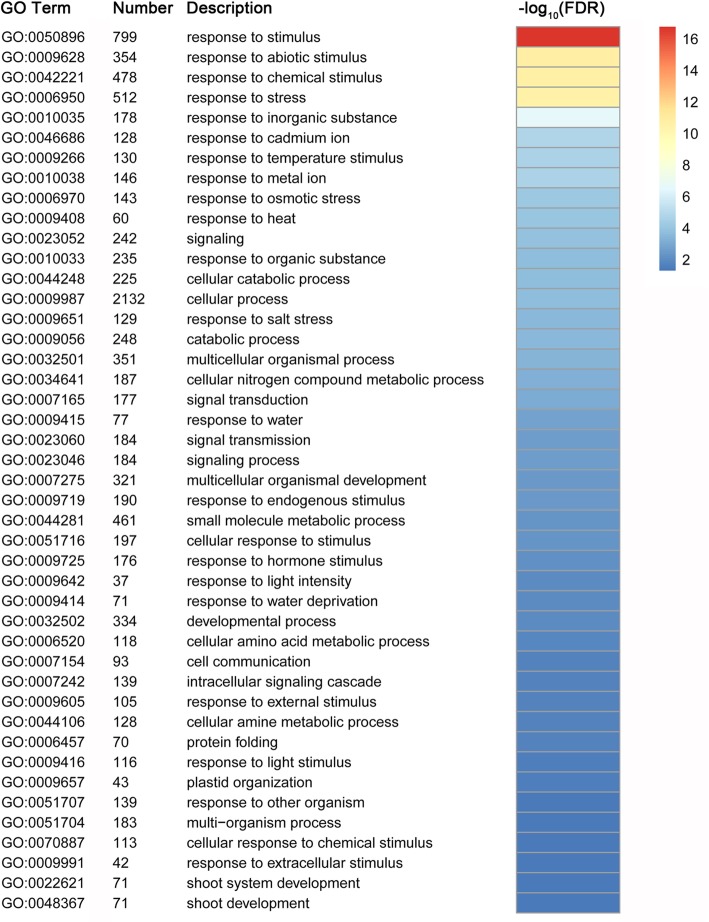
Enrichment of GO biological process terms for the 5400 nonadditive genes under heat treatment

**Fig. 10:**
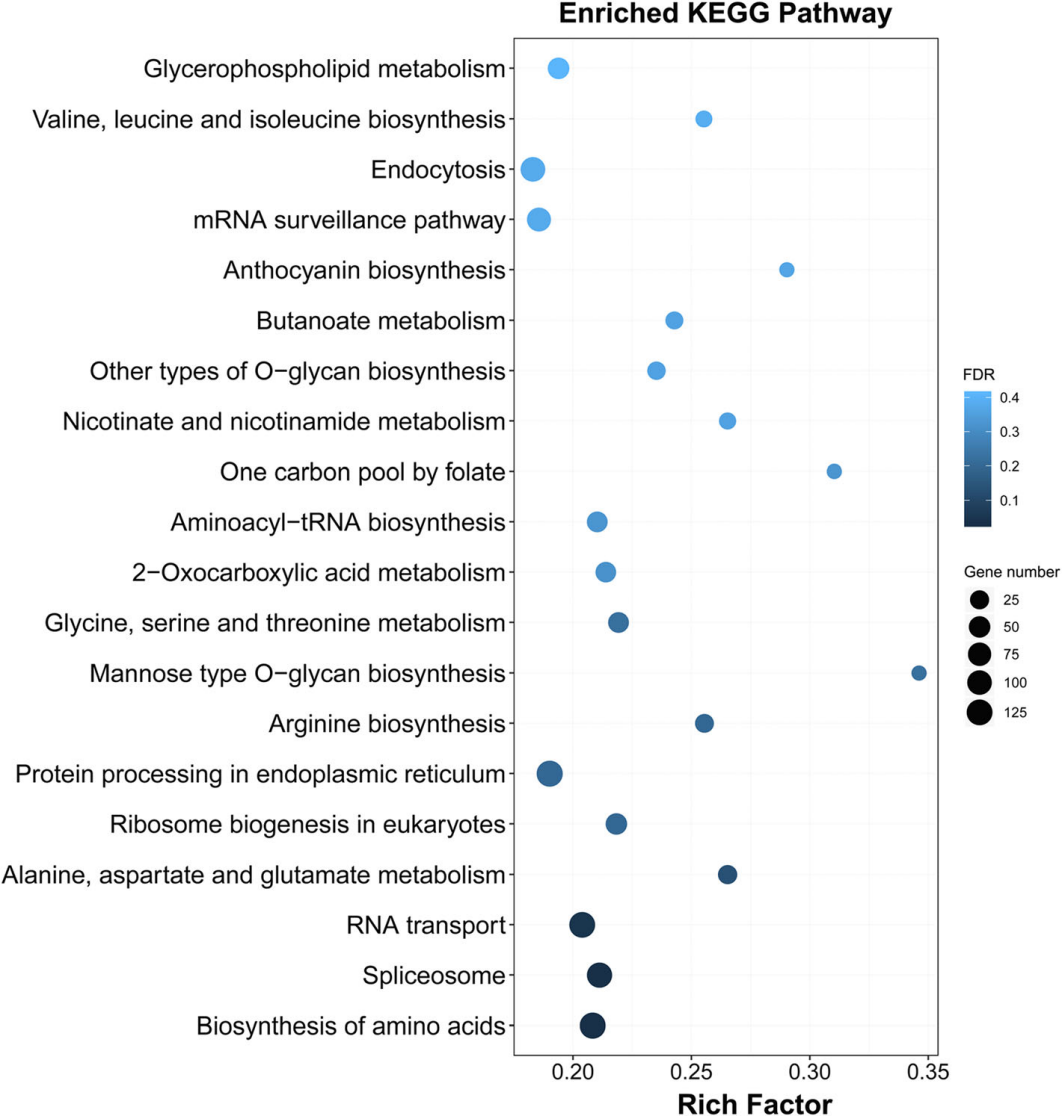
KEGG enrichment analysis of the 5400 nonadditive genes under heat treatment

**Fig. 11:**
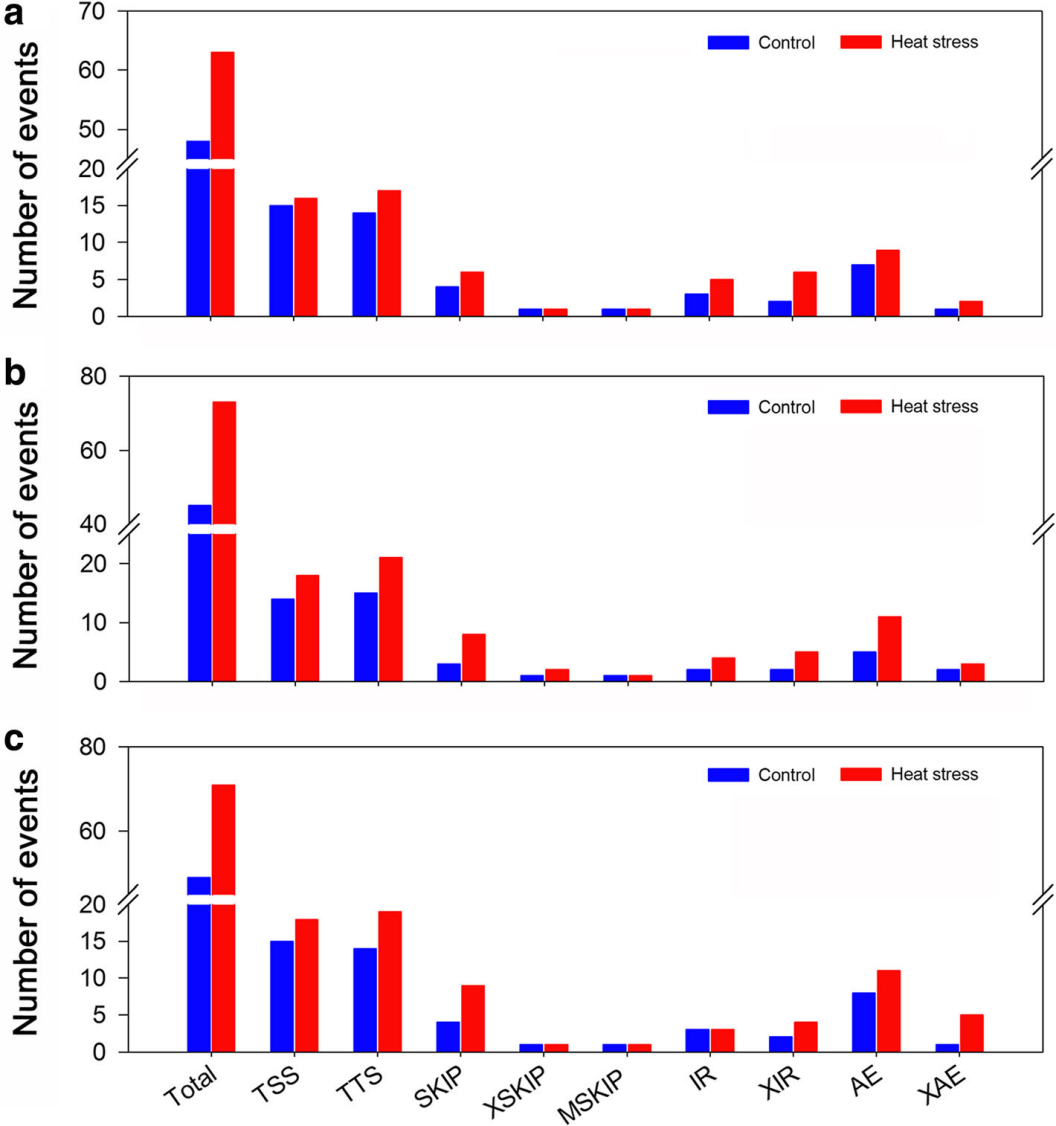
Summary of alternative splicing (AS) events of the 19 genes enriched in the GO term response to heat. **a** AS events in the samples of CR and R; **b** AS events in the samples of CS and S; **c** AS events in the samples of CF_1_ and F_1_. TSS, alternative 5′ first exon (transcription start site); TTS, alternative 3′ last exon (transcription terminal site); SKIP, skipped exon; XSKIP, approximate SKIP; MSKIP, multi-exon SKIP; IR, intron retention; XIR: approximate IR; AE, alternative exon ends; XAE, approximate AE

A total of 3970 and 7653 nonadditive genes were identified from the total expressed genes under the control and heat conditions, respectively. We found that 56.8% (2253 of the 3970 genes) of the nonadditive genes overlapped under control and heat conditions, which suggested that nonadditive gene expression was more conserved in the hybrid under both conditions (Fig. 8b). Such patterns have been observed in previous studies through the comparisons of hybrids and their parents [23]. Among the 7653 nonadditive genes, a total of 5400 genes were found to be specific for heat conditions. GO classification indicated that the 5400 genes were significantly assigned to 44 biological processes (Fig. 9 and Additional file 6 Figure S6). Many genes enriched in the GO terms related to stress responses, such as response to stimulus (GO:0050896), response to abiotic stimulus (GO:0009628), response to chemical stimulus (GO:0042221), response to stress (GO:0006950), and response to osmotic stress (GO:0006970), were overrepresented in all categories. Furthermore, 60 genes were assigned to the GO term response to heat (GO:0009408), of which 33 genes overlapped with the 60 genes that were assigned to the same GO term (response to heat) among the 4518 common DEGs. Functional annotation indicated that most of the 33 genes belonged to heat shock protein and chaperone protein families, suggesting the important roles of these genes in response to heat stress. KEGG pathway analysis revealed that 125, 113, and 125 genes were involved in the biosynthesis of amino acids, spliceosome and RNA transport pathways (FDR ≤ 5%), respectively (Fig. 10). Alternative splicing can generate multiple transcripts from the same gene, which is a common regulatory mechanism required for stress adaptation. Among the 33 overlapping genes, 19 genes were shown to have multiple AS patterns under both control and heat treatment conditions in the three genotypes (Additional file 11 Table S5). The number of AS events under heat stress was significantly higher than that observed under control conditions (Fig. 11), which might suggest that AS of these genes is important in response to heat stress.

## Discussion

Heterosis has been widely exploited in plant breeding for decades due to the superior performance of heterozygous F_1_ hybrids in comparison with their parental lines. Three main genetic models, comprising dominance, overdominance, and epistasis hypotheses, have been proposed to explain heterosis in classical quantitative genetics [4–6, 9]; however, the molecular and genetic mechanisms of this complex biological phenomenon remain poorly understood [3]. Increasing evidence has indicated that differential gene expression between hybrids and their parental lines may be responsible for heterosis [3, 23, 26]. In the present study, RNA-Seq was adopted to investigate the relationship between expression patterns and heterosis in seedlings of the maize hybrid An’nong 591 and its parental lines CB25 and CM1 grown under control and heat stress conditions.

The RNA-Seq analysis showed that 56.2% of the high confidence gene models in the maize reference genome were expressed in at least one of the samples. The high transcriptomic correlation of the three biological replicates of each sample was verified by PCA and Pearson’s correlation analysis, which supported the reproducibility of the RNA-Seq data. Nine pairwise comparisons of gene expression in the three genotypes were performed, and thousands of DEGs were identified for each pair. Under both of control and heat treatment conditions, the maximum number of DEGs was detected between the two parental lines (CS vs. CR and S vs. R). Previous findings have indicated the correlation of gene expression variation and heterosis. About 70% (fold change ≥2 and FDR < 5%: 22.9%) of expressed genes were differentially expressed between maize inbred lines B73 and Mo17, and 42–57% (fold change≥2 and FDR < 5%: 7.6–10.0%) of all expressed genes were differentially expressed between one of the maize hybrids and one of its parents [15]. In rice, the DEGs accounted for 10.6% of the total gene set between the super hybrid LYP9 and its parental cultivars [19]. In the present study, 26.1% of the total expressesd genes were differentially expressed between CB25 and CM1 under control conditions, but 8468 (38.2%) DEGs were identified after heat treatment (Additional file 4 Figure S4), suggesting the difference in gene action of the two inbred lines in response to heat stress. The significant difference in expression between CB25 and CM1 may be an important genetic component responsible for the heterosis of An’nong 591. We also observed that the number of DEGs between the F_1_ hybrid and its maternal line (11.0 and 21.3%) was significantly higher than that between the F_1_ hybrid and its paternal lines (9.7 and 13.6%) in both conditions. These results suggested that gene expression in the hybrid was more similar to the paternal line, especially under heat stress. We concluded that the superior heat tolerance of An’nong 591 was contributed mainly by its paternal line CM1, which was consistent with the evaluation of the phenotypic and physiological characteristics of the three genotypes.

Among the nine pairwise comparisons, the number of DEGs in control versus heat stress pairs was significantly higher than most of the other pairs. The number of up-regulated genes was greater than the number of down-regulated genes in each pair except S vs. R. Previous studies have also reported that up-regulated genes accounted for a larger proportion of the DEGs [23, 45, 46]. For example, more than 60% of DEGs were up-regulated in maize primary roots upon water deficit stress [23]. In this study, the number of up-regulated DEGs 53.80% (14,543 of 27,030 genes) was higher than the down-regulated genes (CF1 vs. F1, CR vs. R, and CS vs. S), suggesting that numerous genes tend to be activated under heat treatment. A total of 4629 common DEGs were identified among the three pairs CF_1_ vs. F_1_, CR vs. R, and CS vs. S to screen for candidate genes involved in the response to heat stress, and 2096 common down-regulated and 2422 common up-regulated genes were further identified among these genes. Functional classification of the 4518 common down- and up-regulated genes was further analyzed by GO enrichment analysis. Many genes were overrepresented in biological process associated with diverse stress response. A total of 60 genes were enriched in the GO term response to heat (GO:0009408), and most of them were significantly up-regulated under heat treatment, which suggested that these common DEGs performed important functions of in response to heat stress. Among the 2096 common down-regulated genes, a total of 119 significant GO terms were enriched. We observed that GO terms associated with photosynthesis, light reaction and stress responses were overrepresented in the biological process category (Additional file 9 Table S3). Only 19 significant biological process GO terms were enriched for the 2422 up-regulated DEGs, and genes enriched in the GO terms were mainly associated with various stress responses (Additional file 10 Table S4). In particular, 53 genes enriched in the term response to heat (GO:0009408), was the most highly represented GO category. These findings indicated the diverse roles of down- and up-regulated genes in response to heat stress.

In the present study, 17.9 and 34.5% of the total expressed genes (22,195) exhibited nonadditive expression under the control and heat stress conditions, respectively. The proportion of nonadditive genes differed notably from that reported in previous studies. For example, 22% of the differentially regulated ESTs were significantly different from the mid-parent value in the Mo17 × B73 maize hybrid [3]. Using the same genotypes, 35 and 27% of genes exhibited nonadditive expression under control and water deficit conditions, respectively [23]. Nonadditive genes accounted for the majority of DEGs (51.2% overdominant, 26% partially dominant and 12.6% dominant) in four genetically unrelated maize inbred lines and their F_1_ crosses [17]. We concluded that the differences among these studies were mainly attributed to different approaches and technical limitations [20]. In the present study, only 17.9% of the total expressed genes displayed expression levels in the hybrid that differed significantly from the mid-parent value under control conditions, which suggested that additive genes have a fundamental role in maize development and heterosis. However, we noted that the number of nonadditive genes under heat treatment was significantly higher than that under the control conditions. In particular, more than 2.5-times the number of underdominant and overdominant genes, respectively, were identified under heat stress conditions, compared with the number of genes identified under control conditions, which suggested nonadditive genes play important roles in the response to heat stress. Nonadditive expression was suggested to be associated with heterosis, and 56.8% of nonadditive genes overlapped between the control and heat stress conditions. Similarly, 47% of the nonadditive genes overlapped between the control and water deficit conditions in a previous study [23]. Together, these results suggest that nonadditive genes show conserved expression in maize hybrids. We observed a total of 5400 nonadditive genes that were particularly associated with heat stress, and GO enrichment analysis indicated that many genes were significantly enriched in biological processes associated with diverse stress responses. Sixty genes were assigned to the GO term response to heat, and 33 genes overlapped with the 60 genes from the common 4518 DEGs that were also enriched in the same GO term. In addition, KEGG pathway analysis indicated that 113 genes were significantly enriched in the spliceosome metabolic pathways. AS is a post-transcriptional regulatory mechanism that allows a single gene to generate multiple transcripts, and has been demonstrated to play important regulatory roles in diverse developmental processes and stress adaptations [47–50]. Consistent with previous studies [51, 52], we found high temperature had an impact on splicing regulation. Among the 33 overlapped genes, 19 genes exhibited a strong AS response, with multiple AS events under heat stress. Of the 19 genes, the majority belonged to heat shock protein and chaperone protein families, which have been demonstrated to play important roles in heat tolerance [53–55]. Thus, these findings also provide a novel strategy to improve plant tolerance to heat stress with alternative transcripts.

## Conclusions

Here, we provide a global view of the transcriptomic divergence of the maize hybrid An’nong 591 and its parental lines under heat stress using RNA-Seq, and nonadditive genes were further analyzed to explore the underlying mechanism of heterosis. Our results reveal the important roles of nonadditive genes in the response to heat stress, and provide new insight into mechanisms of heterosis in heat tolerance in maize hybrid.

## Methods

### Plant material and heat treatment

Seedlings of the maize (*Zea mays* L.) hybrid An’nong 591 (CB25 × CM1) and its parental lines were grown in a greenhouse at 28 °C/23 °C (day/night) with a 16-h light/8-h dark photoperiod. An’nong 591 and its parental lines were generated by Professor Qing Ma and Beijiu Cheng. For heat treatment, seedlings were incubated at 42 °C/35 °C (day/night) for two days when the third leaf was fully expanded. During the period of heat treatment, the seedlings were watered every day, and control plants were maintained under non-stress conditions. After heat treatment, the third leaf was harvested, immediately frozen in liquid nitrogen and stored at − 80 °C for RNA isolation. Before and after heat treatment, the relative water content (RWC) and relative electrolyte leakage (REL) were measured in the seedlings in accordance with methods described previously [34, 35]. Malondialdehyde (MDA) content was measured by a kit according to the instruction manual (Nanjing Jiancheng Bioengineering Institute, China). Statistical analysis of RWC, REL and MDA content was performed using Student’s *t*-test based on three biological replicates. Mid-parent heterosis (MPH) and high-parent heterosis (HPH) were determined using the following formulas: MPH (%) = (F_1_ − MP)/MP and HPH (%) = (F_1_ − HP)/HP, where MP is the average value of the two parents, and HP is the highest value of the two parents.

### RNA isolation and cDNA library construction

Total RNA was extracted using TRIzol reagent (Invitrogen, USA) according to the manufacturer’s protocol. The quality and purity of RNAs was assessed by Agilent 2100 Bioanalyzer (Agilent Technologies, CA), NanoDrop spectrophotometer (Thermo Fisher Scientific Inc., USA) and 1% agarose gel. Total RNA (1 μg) with RNA integrity number (RIN) values above 7 was used for library construction of each sample. Poly (A)-containing RNA was purified from total RNA using the NEBNext® Poly (A) mRNA Magnetic Isolation Module (New England Biolabs Inc., USA). The cDNA library preparations were constructed using a NEBNext® Ultra™ RNA Library Prep Kit for Illumina® (New England Biolabs Inc., USA) according to the manufacturer’s protocol. The libraries with different indices were loaded onto an Illumina HiSeq X Ten platform for sequencing, which generated 2 × 150 bp paired-end reads. The raw reads were submitted to the GenBank GEO database under accession number GSE122866 (https://www.ncbi.nlm.nih.gov/geo/query/acc.cgi?acc=GSE122866).

### Sequence assembly and data analysis

Raw reads were filtered to obtained high-quality reads by removing reads containing adapter or poly (N) sequences, low quality sequences (Q < 20) from the 5′ and 3′ ends of the reads, and sequences shorter than 75 bp. Clean reads (the remaining high-quality reads) were subsequently aligned to the maize B73 reference genome (RefGen_v3) using HISAT software (v2.0.1) [36]. HTSeq software (v0.6.1) was used to count the read numbers mapped to each gene [37]. The gene expression level was measured in terms of the fragments per kilobase of transcript per million mapped reads (FPKM) [38]. Novel transcripts and multiple transcripts generated by alternative splicing (AS) were assembled using StringTie software (v1.0.4) [39]. The DEGs between the two sets of samples were identified using the DESeq2 package (1.24.0) [40], and the resulting values were adjusted using the Benjamini and Hochberg approach for controlling the false discovery rate (FDR) [41]. Only genes with fold changes ≥2 and FDR ≤ 5% were determined to be significantly differentially expressed. The DEGs in common between different samples were identified by venn diagram analysis using the web-based tool (http://bioinformatics.psb.ugent.be/webtools/Venn/). The gene expression levels of DEGs were transformed by log_10_ (FPKM + 1), and used to draw a heat map with R package pheatmap.

### Validation of RNA-Seq by quantitative real-time PCR

Quantitative real-time polymerase chain reaction (qRT-PCR) was used to validate the gene expression levels of DEGs detected by RNA-Seq. Total RNA from the same samples as used for cDNA library construction was used for first-strand cDNA synthesis with the PrimeScript™ RT reagent kit with gDNA Eraser (TaKaRa, China). Primers were designed using Primer Express 3.0 software (Applied Biosystems, USA) and are listed in Additional file 7 Table S1. The qRT-PCR was performed using a Roche LightCycler480 II real-time PCR system (Roche, Germany). Each reaction contained 10 μL of 2 × FastStart Universal SYBR Green Master (Roche, Germany), 2.0 μL diluted cDNA, and 0.8 μL each of the forward and reverse primers in a final volume of 20 μL. The PCR conditions consisted of pre-denaturation at 95 °C for 5 min, followed by 40 cycles of 95 °C for 15 s, 60 °C for 25 s, and 72 °C for 25 s. At the end of the PCR cycles, melting curve analysis was performed to validate the specificity of the PCR product. The maize *GAPDH* gene (accession number: NM_001111943.1) was used as an internal control for normalization, and three technical replicates of each cDNA sample were performed for qRT-PCR analysis. Data analysis was performed as reported previously [42].

### Gene ontology and pathway enrichment analysis

Gene Ontology (GO) enrichment analysis was performed using agriGO (http://systemsbiology.cau.edu.cn/agriGOv2/) [43]. GO terms with FDR ≤ 5% were considered to be significantly enriched, and were categorized into three types of functional classification, namely cellular component, molecular function and biological process. The significantly enriched GO terms of biological process were used to draw a heat map by transforming FDR values into −log_10_ (FDR) with the R package pheatmap. Pathway analysis was performed using the Kyoto Encyclopedia of Genes and Genomes (KEGG) web server (http://www.kegg.jp/) [44]. Pathways with FDR ≤ 5% were considered to be significantly enriched.

### Identification of nonadditive genes

To identify nonadditive genes, the gene expression patterns that differed between the hybrid and its parental lines (CB25 and CM1) were analyzed by comparing the average expression values of all three biological replicates of the hybrids with the mid-parent values of the parental lines under the control conditions and heat stress treatment. Genes with FDR ≤ 5% were considered to be nonadditively expressed. According to the method described previously [3], the nonadditive genes were further divided into different categories, including high-parent dominance, low-parent dominance, overdominance, and underdominance.

## Additional files

Additional file 1: **Figure S1.** Comparison of expression values of the three genotypes under the control and heat treatment conditions. Expression values were transformed by log_10_ (FPKM). The horizontal line represents the median of each replicate of the three genotypes. (TIF 10316 kb)

Additional file 2: **Figure S2.** Pearson’s correlation analysis of the biological replicates of each genotype. (TIF 6944 kb)

Additional file 3: **Figure S3.** Heatmap of the differentially expressed genes under the control and heat treatment conditions. (TIF 7379 kb)

Additional file 4: **Figure S4.** Numbers of differentially expressed genes between the hybrid and its parents. **a** Total number of DEGs under the control conditions. **b** Total number of DEGs under the heat treatment. **c** Total number of DEGs between the control and heat treatment conditions. **d** Venn diagram of common DEGs under the control conditions. **e** Venn diagram of common DEGs under the heat treatment. **f** Venn diagram of common DEGs between the control and heat treatment conditions. (TIF 12294 kb)

Additional file 5: **Figure S5.** Hierarchical tree graphs of enriched GO terms in the biological process category for the 4518 common DEGs. Hierarchical tree graph were generated using agriGO. The GO ID (adjusted *P* values), term definition, and statistical information are shown in the boxes. Significant terms (adjusted *P* ≤ 0.05) are in colored boxes, and GO terms with non-significant are in white boxes. Solid, dashed, and dotted lines in the graphs represent two, one and zero enriched terms at both ends connected by the line. (TIF 16606 kb)

Additional file 6: **Figure S6.** Hierarchical tree graph of enriched GO terms in the biological process category for the 5400 nonadditive genes. (TIF 16250 kb)

Additional file 7: **Table S1.** Gene-specific primers used for qRT-PCR analysis. (XLS 30 kb)

Additional file 8: **Table S2.** Detailed information of the RNA-Seq data. (DOCX 22 kb)

Additional file 9: **Table S3.** Significantly enriched GO biological process terms for 2096 common down-regulated differentially expressed genes. (DOCX 22 kb)

Additional file 10: **Table S4.** Significantly enriched GO biological process terms for 2422 common up-regulated differentially expressed genes. (DOCX 16 kb)

Additional file 11: **Table S5.** Comparative analysis of alternative splicing events of the 19 genes enriched in the GO term response to heat. (XLSX 14 kb)

## Data Availability

The raw data supporting the conclusions in this article are available in the GenBank GEO database under accession number GSE122866 (https://www.ncbi.nlm.nih.gov/geo/query/acc.cgi?acc=GSE122866).

## Abbreviations

AS: Alternative splicing
BPH: Best-parent heterosis
DEGs: Differentially expressed genes
FDR: False discovery rate
FPKM: Fragments per kilobase of transcript per million mapped reads
GO: Gene ontology
KEGG: Kyoto encyclopedia of genes and genomes
MDA: Malondialdehyde
MPH: Mid-parent heterosis
PCA: Principal component analysis
PCR: Polymerase chain reaction
qRT-PCR: Quantitative real-time PCR
REL: Relative electrolyte leakage
RNA-Seq: RNA sequencing
RWC: Relative water content
