# Supplementary figures and images for: Comparative transcriptome analysis reveals important roles of nonadditive genes in maize hybrid An’nong 591 under heat stress
Source: BMC Plant Biol. 2019 Jun 24;19:273. doi: 10.1186/s12870-019-1878-8 (PMC6591960; doi:10.1186/s12870-019-1878-8)

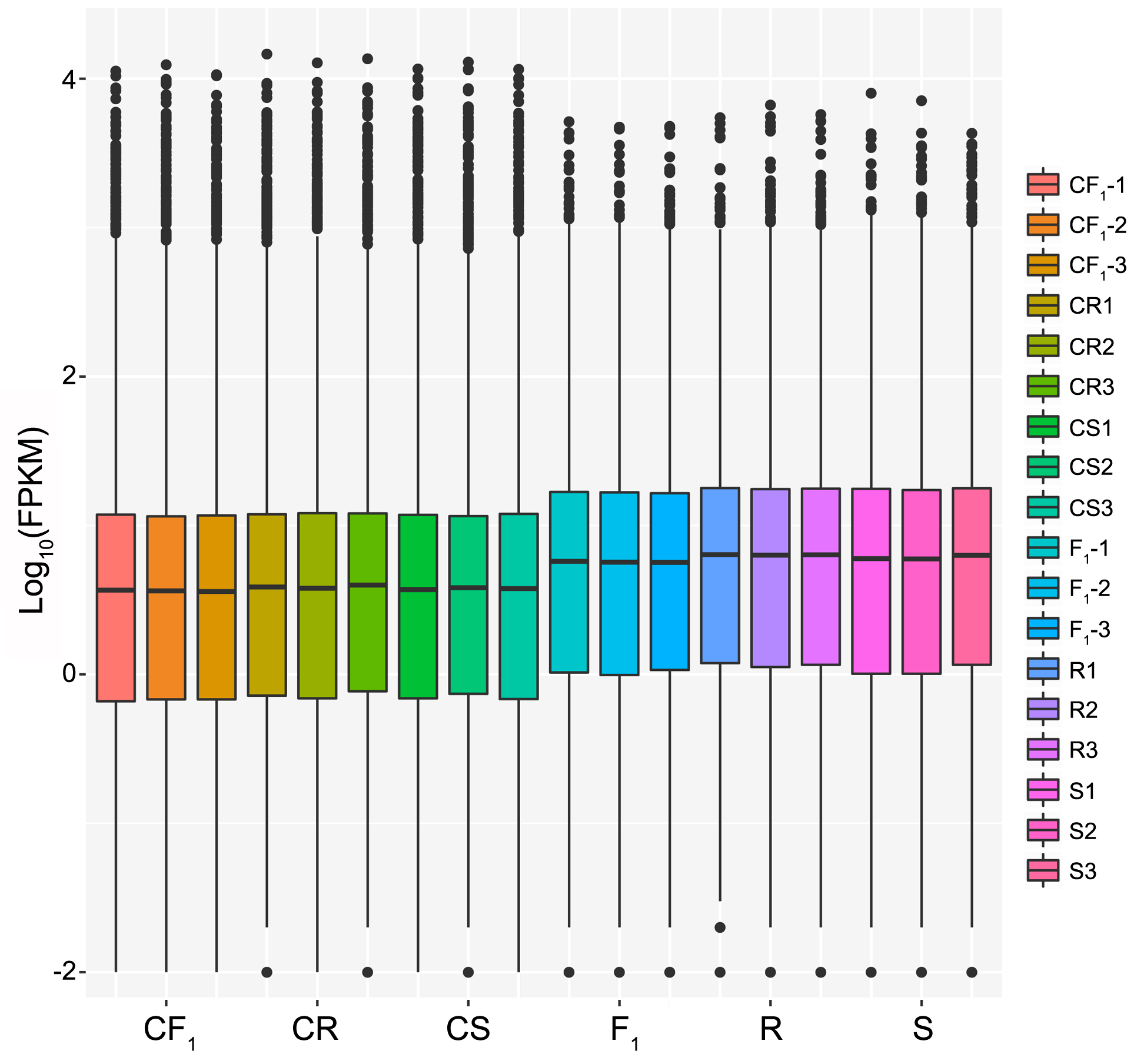

Supplement: Supplementary file 1 — Figure S1. Comparison of expression values of the three genotypes under the control and heat treatment conditions. Expression values were transformed by log10 (FPKM). The horizontal line represents the median of each replicate of the three genotypes. (TIF 10316 kb) [file 12870_2019_1878_MOESM1_ESM.tif]

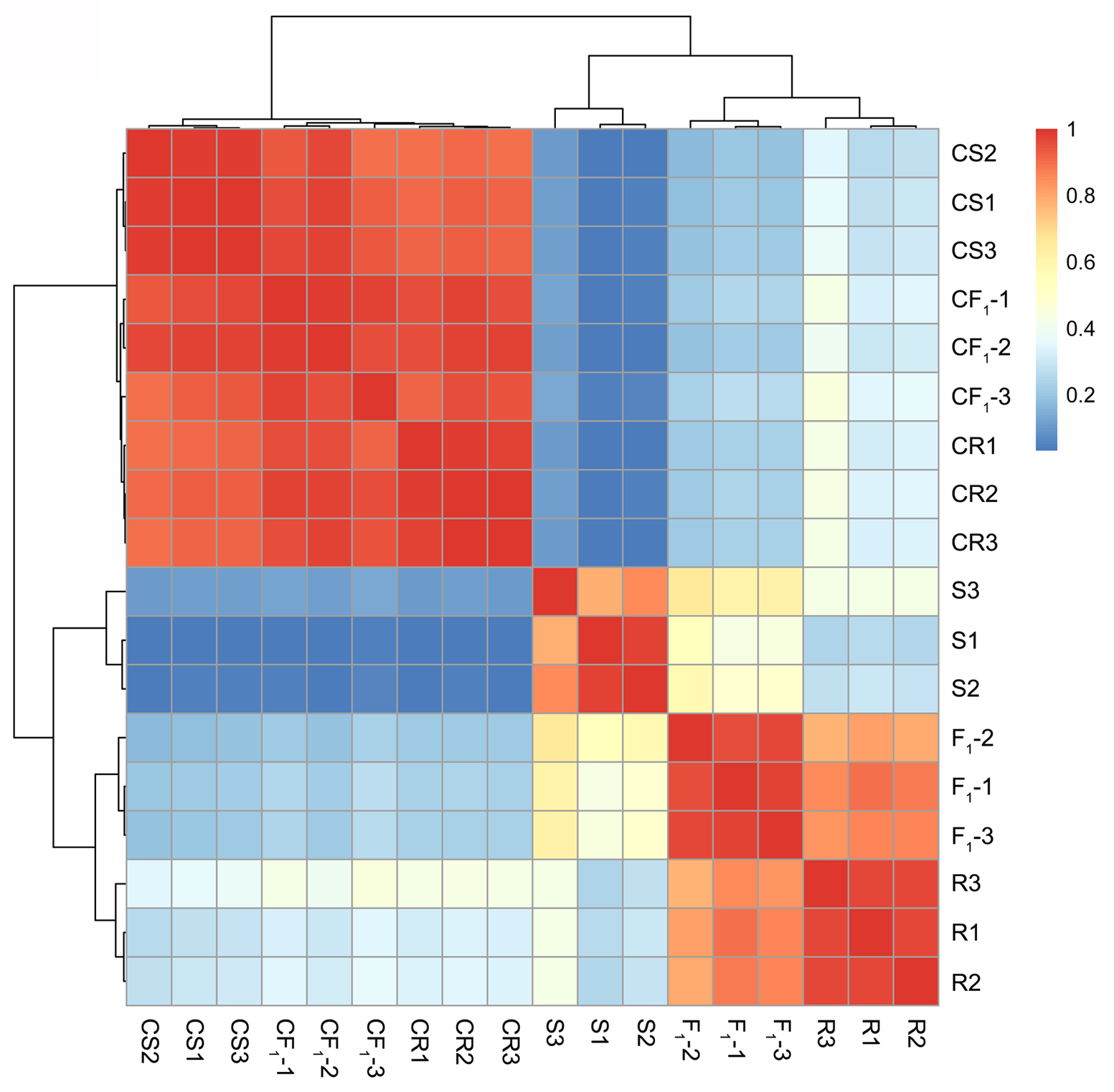

Supplement: Supplementary file 2 — Figure S2. Pearson’s correlation analysis of the biological replicates of each genotype. (TIF 6944 kb) [file 12870_2019_1878_MOESM2_ESM.tif]

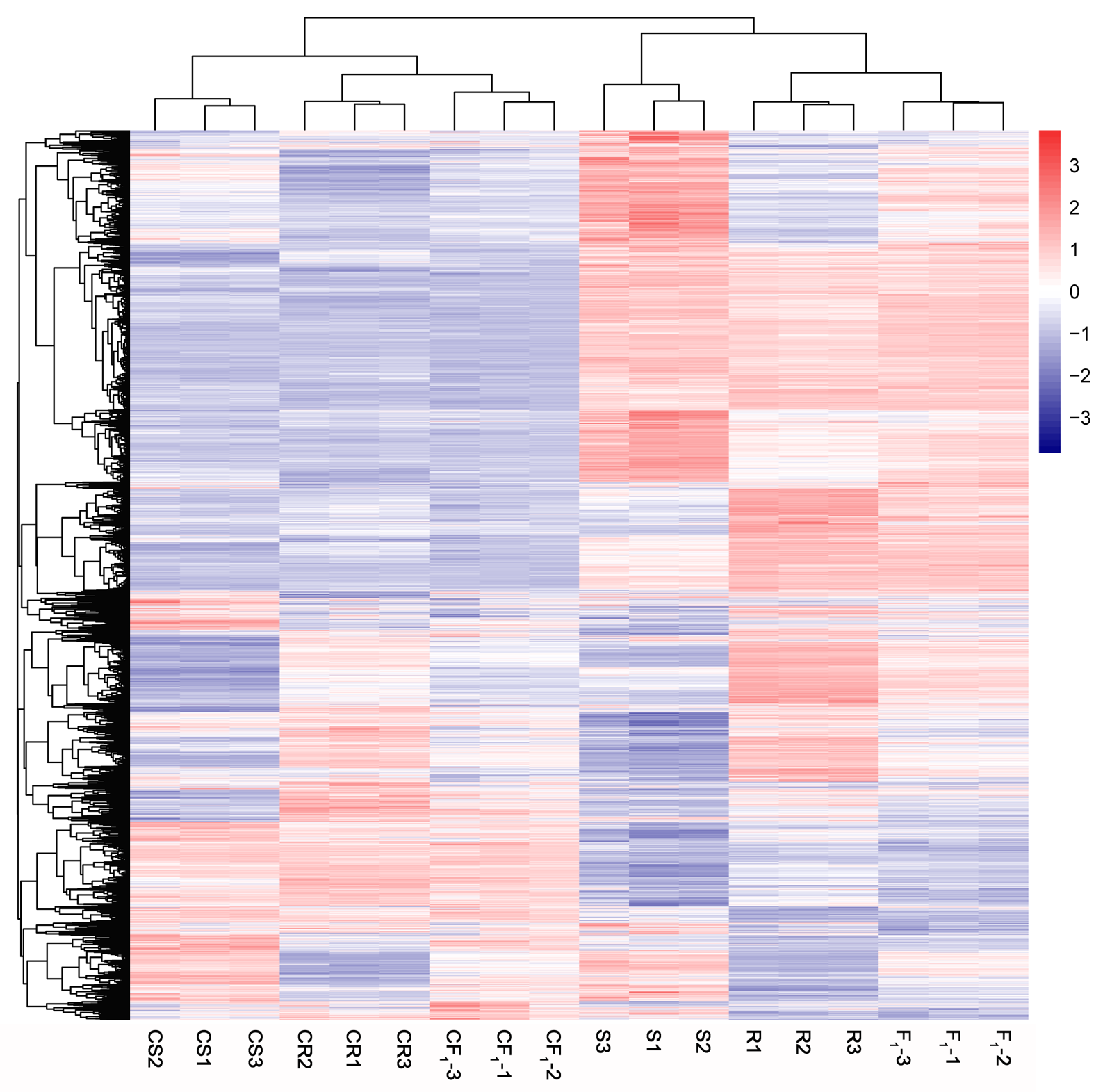

Supplement: Supplementary file 3 — Figure S3. Heatmap of the differentially expressed genes under the control and heat treatment conditions. (TIF 7379 kb) [file 12870_2019_1878_MOESM3_ESM.tif]

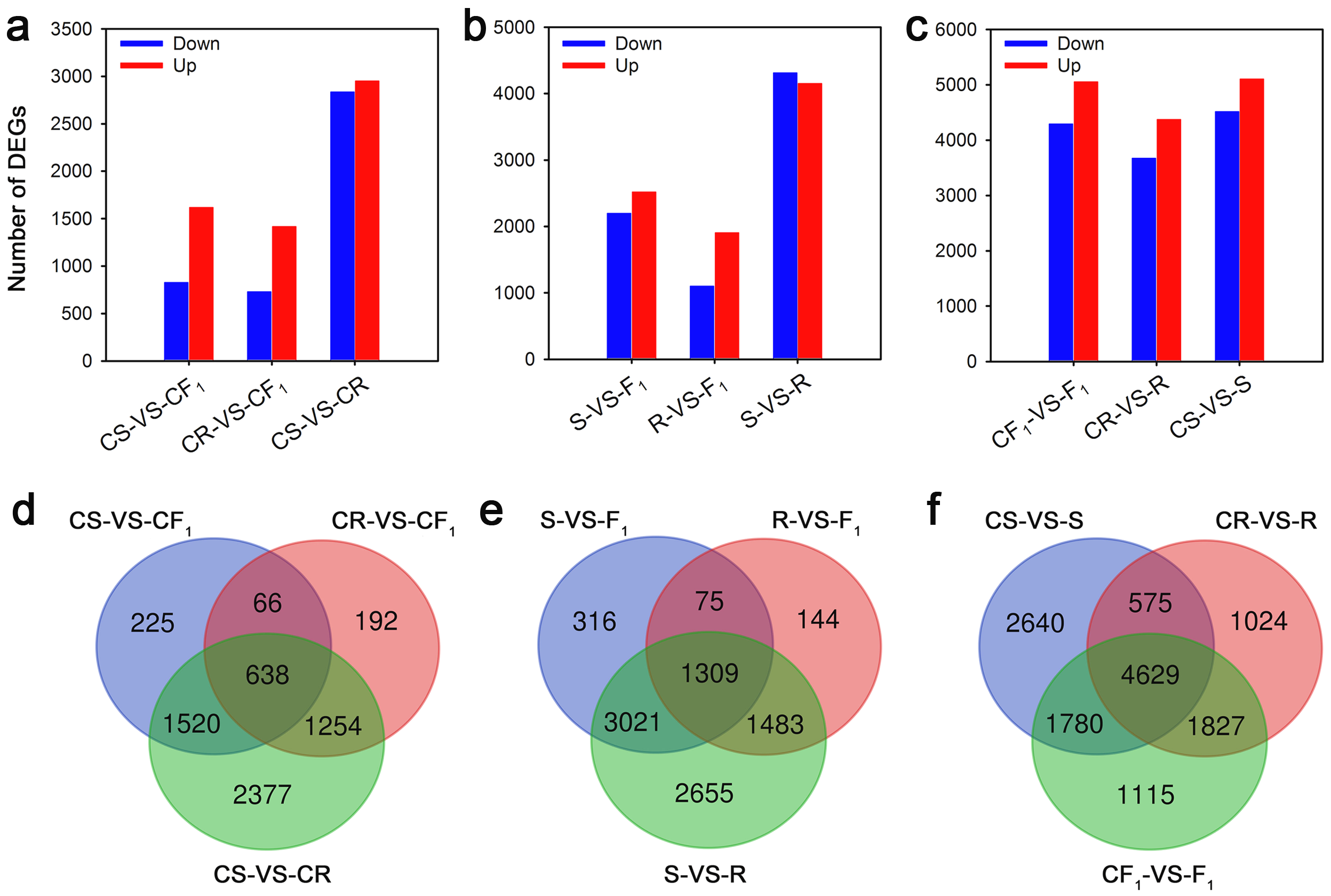

Supplement: Supplementary file 4 — Figure S4. Numbers of differentially expressed genes between the hybrid and its parents. a Total number of DEGs under the control conditions. b Total number of DEGs under the heat treatment. c Total number of DEGs between the control and heat treatment conditions. d Venn diagram of common DEGs under the control conditions. e Venn diagram of common DEGs under the heat treatment. f Venn diagram of common DEGs between the control and heat treatment conditions. (TIF 12294 kb) [file 12870_2019_1878_MOESM4_ESM.tif]

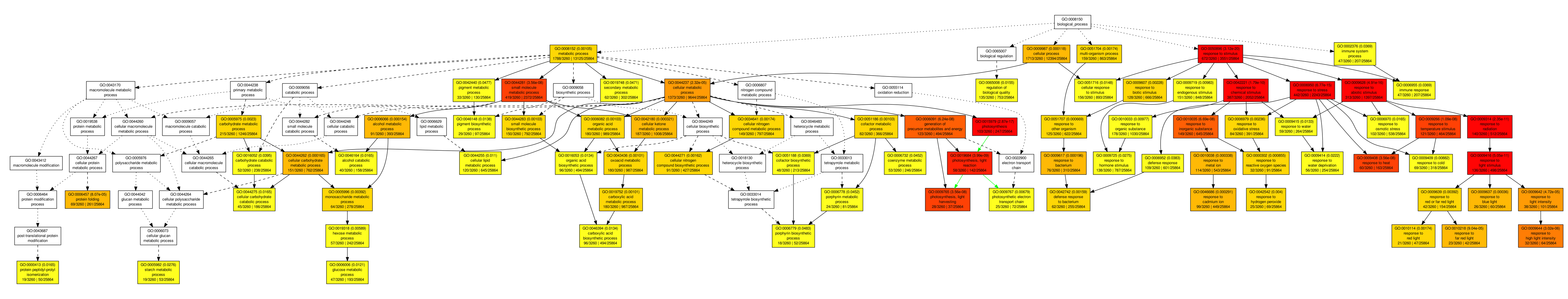

Supplement: Supplementary file 5 — Figure S5. Hierarchical tree graphs of enriched GO terms in the biological process category for the 4518 common DEGs. Hierarchical tree graph were generated using agriGO. The GO ID (adjusted P values), term definition, and statistical information are shown in the boxes. Significant terms (adjusted P ≤ 0.05) are in colored boxes, and GO terms with non-significant are in white boxes. Solid, dashed, and dotted lines in the graphs represent two, one and zero enriched terms at both ends connected by the line. (TIF 16606 kb) [file 12870_2019_1878_MOESM5_ESM.tif]

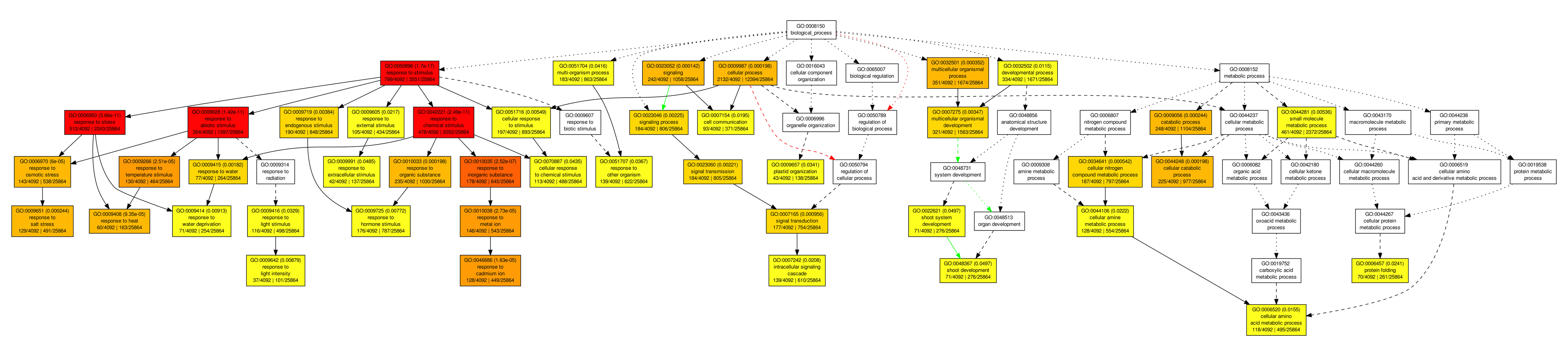

Supplement: Supplementary file 6 — Figure S6. Hierarchical tree graph of enriched GO terms in the biological process category for the 5400 nonadditive genes. (TIF 16250 kb) [file 12870_2019_1878_MOESM6_ESM.tif]
