# Supplementary material for: Comparative transcriptome analysis reveals important roles of nonadditive genes in maize hybrid An’nong 591 under heat stress
Source: BMC Plant Biol. 2019 Jun 24;19:273. doi: 10.1186/s12870-019-1878-8 (PMC6591960; doi:10.1186/s12870-019-1878-8)
Supplement: Supplementary file 8 — Table S2. Detailed information of the RNA-Seq data. (DOCX 22 kb) [file 12870_2019_1878_MOESM8_ESM.docx]

Table S2. Detailed information of the RNA-Seq data.

| **Sample name** | **Genotype** | **Biological replicate** | **Raw reads** | **Clean reads** | **Base of** **clean reads (bp)** | **Q20 (%)** | **Q30 (%)** | **GC (%)** | **Total mapped on genome (%)** | **Uniquely mapped on genome (%)** |
| --- | --- | --- | --- | --- | --- | --- | --- | --- | --- | --- |
| CF_1_ | F_1_ hybrid | 1 | 46,410,984 | 46,162,994 | 6,870,384,188 | 97.06 | 92.45 | 57.37 | 81.98 | 74.44 |
| CF_1_ | F_1_ hybrid | 2 | 43,190,978 | 42,978,124 | 6,400,947,489 | 97.03 | 92.36 | 58.27 | 81.95 | 74.19 |
| CF_1_ | F_1_ hybrid | 3 | 39,452,718 | 39,199,660 | 5,838,062,439 | 97.19 | 92.77 | 57.99 | 81.98 | 75.20 |
| CR | Paternal line | 1 | 49,067,710 | 48,811,462 | 7,267,513,530 | 97.18 | 92.73 | 57.42 | 82.61 | 74.95 |
| CR | Paternal line | 2 | 49,879,168 | 49,634,050 | 7,391,352,760 | 97.19 | 92.72 | 57.43 | 82.90 | 75.53 |
| CR | Paternal line | 3 | 45,251,360 | 44,957,732 | 6,694,502,566 | 97.14 | 92.66 | 57.79 | 82.74 | 75.44 |
| CS | Maternal line | 1 | 49,534,704 | 49,289,866 | 7,338,489,345 | 97.17 | 92.69 | 58.00 | 81.86 | 73.72 |
| CS | Maternal line | 2 | 45,860,086 | 45,650,454 | 6,797,706,710 | 97.19 | 92.68 | 58.74 | 81.24 | 72.76 |
| CS | Maternal line | 3 | 46,304,178 | 46,083,168 | 6,862,429,223 | 97.05 | 92.4 | 57.82 | 81.40 | 73.41 |
| F_1_ | F_1_ hybrid | 1 | 48,842,088 | 48,591,188 | 7,231,695,501 | 97.24 | 92.93 | 54.04 | 80.40 | 75.00 |
| F_1_ | F_1_ hybrid | 2 | 49,489,088 | 49,263,924 | 7,333,088,030 | 97.27 | 92.99 | 54.11 | 80.10 | 74.66 |
| F_1_ | F_1_ hybrid | 3 | 47,825,846 | 47,531,426 | 7,075,983,902 | 97.37 | 93.26 | 54.44 | 80.45 | 75.09 |
| R | Paternal line | 1 | 42,780,020 | 42,560,756 | 6,335,233,774 | 97.14 | 92.71 | 54.3 | 80.55 | 75.12 |
| R | Paternal line | 2 | 49,642,118 | 49,382,198 | 7,351,633,308 | 97.15 | 92.75 | 54.23 | 81.10 | 75.50 |
| R | Paternal line | 3 | 42,555,924 | 42,292,366 | 6,298,055,603 | 97.14 | 92.74 | 54.84 | 81.13 | 75.65 |
| S | Maternal line | 1 | 47,527,996 | 47,275,864 | 7,035,396,918 | 97.24 | 92.93 | 54.32 | 77.90 | 72.57 |
| S | Maternal line | 2 | 41,986,560 | 41,788,194 | 6,218,459,447 | 97.16 | 92.74 | 54.15 | 78.12 | 72.75 |
| S | Maternal line | 3 | 43,319,226 | 43,038,866 | 6,407,599,768 | 97.18 | 92.85 | 54.81 | 78.54 | 73.18 |
