# Supplementary material for: Comparative transcriptome analysis reveals important roles of nonadditive genes in maize hybrid An’nong 591 under heat stress
Source: BMC Plant Biol. 2019 Jun 24;19:273. doi: 10.1186/s12870-019-1878-8 (PMC6591960; doi:10.1186/s12870-019-1878-8)
Supplement: Supplementary file 9 — Table S3. Significantly enriched GO biological process terms for 2096 common down-regulated differentially expressed genes. (DOCX 22 kb) [file 12870_2019_1878_MOESM9_ESM.docx]

Table S3. Significantly enriched GO biological process terms for 2,096 common down-regulated differentially expressed genes.

| **GO term** | **Description** | **Gene number** | **FDR** |
| --- | --- | --- | --- |
| GO:0015979 | photosynthesis | 101 | 3.40E-38 |
| GO:0019684 | photosynthesis, light reaction | 56 | 9.30E-20 |
| GO:0006091 | generation of precursor metabolites and energy | 100 | 7.90E-18 |
| GO:0009765 | photosynthesis, light harvesting | 28 | 1.30E-14 |
| GO:0050896 | response to stimulus | 356 | 4.30E-14 |
| GO:0009416 | response to light stimulus | 90 | 1.90E-13 |
| GO:0009314 | response to radiation | 91 | 2.70E-13 |
| GO:0009628 | response to abiotic stimulus | 172 | 3.90E-12 |
| GO:0009987 | cellular process | 932 | 6.50E-09 |
| GO:0051186 | cofactor metabolic process | 64 | 8.60E-09 |
| GO:0044237 | cellular metabolic process | 752 | 9.00E-09 |
| GO:0008152 | metabolic process | 976 | 1.00E-08 |
| GO:0044281 | small molecule metabolic process | 235 | 3.30E-08 |
| GO:0009617 | response to bacterium | 56 | 3.80E-08 |
| GO:0010218 | response to far red light | 19 | 4.60E-07 |
| GO:0051707 | response to other organism | 83 | 1.00E-06 |
| GO:0006950 | response to stress | 216 | 1.40E-06 |
| GO:0042742 | defense response to bacterium | 46 | 1.40E-06 |
| GO:0010114 | response to red light | 19 | 1.60E-06 |
| GO:0009767 | photosynthetic electron transport chain | 23 | 1.60E-06 |
| GO:0051188 | cofactor biosynthetic process | 41 | 1.60E-06 |
| GO:0009637 | response to blue light | 21 | 1.80E-06 |
| GO:0009607 | response to biotic stimulus | 85 | 2.90E-06 |
| GO:0009639 | response to red or far red light | 33 | 4.50E-06 |
| GO:0046148 | pigment biosynthetic process | 25 | 1.10E-05 |
| GO:0006778 | porphyrin metabolic process | 22 | 2.90E-05 |
| GO:0055114 | oxidation reduction | 184 | 2.90E-05 |
| GO:0042221 | response to chemical stimulus | 193 | 3.10E-05 |
| GO:0042440 | pigment metabolic process | 28 | 3.90E-05 |
| GO:0033013 | tetrapyrrole metabolic process | 22 | 7.00E-05 |
| GO:0044262 | cellular carbohydrate metabolic process | 88 | 7.00E-05 |
| GO:0006779 | porphyrin biosynthetic process | 17 | 7.10E-05 |
| GO:0006952 | defense response | 73 | 0.00012 |
| GO:0044255 | cellular lipid metabolic process | 76 | 0.00019 |
| GO:0006732 | coenzyme metabolic process | 39 | 0.00019 |
| GO:0042180 | cellular ketone metabolic process | 106 | 0.00023 |
| GO:0051704 | multi-organism process | 94 | 0.00023 |
| GO:0033014 | tetrapyrrole biosynthetic process | 17 | 0.00031 |
| GO:0006811 | ion transport | 72 | 0.0005 |
| GO:0009657 | plastid organization | 26 | 0.00064 |
| GO:0006066 | alcohol metabolic process | 51 | 0.00079 |
| GO:0034641 | cellular nitrogen compound metabolic process | 86 | 0.00079 |
| GO:0055085 | transmembrane transport | 89 | 0.00079 |
| GO:0044271 | cellular nitrogen compound biosynthetic process | 54 | 0.00083 |
| GO:0005996 | monosaccharide metabolic process | 40 | 0.00087 |
| GO:0019748 | secondary metabolic process | 42 | 0.0011 |
| GO:0019318 | hexose metabolic process | 36 | 0.0012 |
| GO:0006721 | terpenoid metabolic process | 20 | 0.0012 |
| GO:0006006 | glucose metabolic process | 31 | 0.0012 |
| GO:0015994 | chlorophyll metabolic process | 14 | 0.0013 |
| GO:0043436 | oxoacid metabolic process | 100 | 0.0013 |
| GO:0019752 | carboxylic acid metabolic process | 100 | 0.0013 |
| GO:0006082 | organic acid metabolic process | 100 | 0.0014 |
| GO:0044275 | cellular carbohydrate catabolic process | 30 | 0.0014 |
| GO:0016114 | terpenoid biosynthetic process | 17 | 0.0014 |
| GO:0015995 | chlorophyll biosynthetic process | 10 | 0.0017 |
| GO:0022900 | electron transport chain | 31 | 0.002 |
| GO:0032787 | monocarboxylic acid metabolic process | 53 | 0.0024 |
| GO:0045087 | innate immune response | 29 | 0.0026 |
| GO:0005982 | starch metabolic process | 14 | 0.0029 |
| GO:0005975 | carbohydrate metabolic process | 118 | 0.003 |
| GO:0044283 | small molecule biosynthetic process | 82 | 0.0031 |
| GO:0046164 | alcohol catabolic process | 26 | 0.0031 |
| GO:0016052 | carbohydrate catabolic process | 34 | 0.0031 |
| GO:0002376 | immune system process | 31 | 0.0031 |
| GO:0006955 | immune response | 31 | 0.0031 |
| GO:0006720 | isoprenoid metabolic process | 23 | 0.0031 |
| GO:0009611 | response to wounding | 30 | 0.0032 |
| GO:0009773 | photosynthetic electron transport in photosystem I | 8 | 0.0035 |
| GO:0006766 | vitamin metabolic process | 18 | 0.0035 |
| GO:0010196 | nonphotochemical quenching | 6 | 0.0037 |
| GO:0044249 | cellular biosynthetic process | 358 | 0.0038 |
| GO:0009409 | response to cold | 41 | 0.004 |
| GO:0009058 | biosynthetic process | 370 | 0.0045 |
| GO:0008610 | lipid biosynthetic process | 54 | 0.0049 |
| GO:0044282 | small molecule catabolic process | 38 | 0.0049 |
| GO:0008299 | isoprenoid biosynthetic process | 20 | 0.006 |
| GO:0046351 | disaccharide biosynthetic process | 14 | 0.0069 |
| GO:0019320 | hexose catabolic process | 24 | 0.0078 |
| GO:0046365 | monosaccharide catabolic process | 24 | 0.0078 |
| GO:0006007 | glucose catabolic process | 24 | 0.0078 |
| GO:0006820 | anion transport | 19 | 0.0079 |
| GO:0009108 | coenzyme biosynthetic process | 21 | 0.0091 |
| GO:0009312 | oligosaccharide biosynthetic process | 14 | 0.01 |
| GO:0051179 | localization | 197 | 0.011 |
| GO:0018130 | heterocycle biosynthetic process | 25 | 0.012 |
| GO:0019252 | starch biosynthetic process | 8 | 0.013 |
| GO:0010027 | thylakoid membrane organization | 9 | 0.014 |
| GO:0009668 | plastid membrane organization | 9 | 0.014 |
| GO:0009110 | vitamin biosynthetic process | 16 | 0.016 |
| GO:0032544 | plastid translation | 6 | 0.017 |
| GO:0009814 | defense response, incompatible interaction | 20 | 0.017 |
| GO:0006096 | glycolysis | 19 | 0.018 |
| GO:0006000 | fructose metabolic process | 7 | 0.02 |
| GO:0009605 | response to external stimulus | 48 | 0.021 |
| GO:0010035 | response to inorganic substance | 65 | 0.024 |
| GO:0006812 | cation transport | 53 | 0.025 |
| GO:0006541 | glutamine metabolic process | 7 | 0.025 |
| GO:0006629 | lipid metabolic process | 92 | 0.025 |
| GO:0046483 | heterocycle metabolic process | 70 | 0.029 |
| GO:0042373 | vitamin K metabolic process | 5 | 0.03 |
| GO:0042371 | vitamin K biosynthetic process | 5 | 0.03 |
| GO:0005986 | sucrose biosynthetic process | 5 | 0.03 |
| GO:0045426 | quinone cofactor biosynthetic process | 7 | 0.03 |
| GO:0006084 | acetyl-CoA metabolic process | 11 | 0.036 |
| GO:0051234 | establishment of localization | 188 | 0.036 |
| GO:0046686 | response to cadmium ion | 48 | 0.037 |
| GO:0016051 | carbohydrate biosynthetic process | 37 | 0.038 |
| GO:0009682 | induced systemic resistance | 6 | 0.038 |
| GO:0005984 | disaccharide metabolic process | 17 | 0.039 |
| GO:0032268 | regulation of cellular protein metabolic process | 15 | 0.039 |
| GO:0071214 | cellular response to abiotic stimulus | 14 | 0.044 |
| GO:0010033 | response to organic substance | 93 | 0.044 |
| GO:0006108 | malate metabolic process | 7 | 0.044 |
| GO:0006739 | NADP metabolic process | 10 | 0.044 |
| GO:0051246 | regulation of protein metabolic process | 16 | 0.044 |
| GO:0009627 | systemic acquired resistance | 10 | 0.044 |
| GO:0010109 | regulation of photosynthesis | 8 | 0.045 |
| GO:0080134 | regulation of response to stress | 16 | 0.047 |
