# Supplementary material for: Comparative transcriptome analysis reveals important roles of nonadditive genes in maize hybrid An’nong 591 under heat stress
Source: BMC Plant Biol. 2019 Jun 24;19:273. doi: 10.1186/s12870-019-1878-8 (PMC6591960; doi:10.1186/s12870-019-1878-8)
Supplement: Supplementary file 10 — Table S4. Significantly enriched GO biological process terms for 2422 common up-regulated differentially expressed genes. (DOCX 16 kb) [file 12870_2019_1878_MOESM10_ESM.docx]

Table S4. Significantly enriched GO biological process terms for 2,422 common up-regulated differentially expressed genes.

| **GO term** | **Description** | **Gene number** | **FDR** |
| --- | --- | --- | --- |
| GO:0009408 | response to heat | 53 | 8.20E-16 |
| GO:0006457 | protein folding | 55 | 6.60E-10 |
| GO:0006950 | response to stress | 226 | 5.80E-09 |
| GO:0009266 | response to temperature stimulus | 73 | 1.70E-08 |
| GO:0050896 | response to stimulus | 316 | 1.10E-07 |
| GO:0010035 | response to inorganic substance | 84 | 1.50E-06 |
| GO:0009644 | response to high light intensity | 22 | 1.70E-06 |
| GO:0042221 | response to chemical stimulus | 194 | 9.00E-06 |
| GO:0042542 | response to hydrogen peroxide | 21 | 1.70E-05 |
| GO:0009628 | response to abiotic stimulus | 141 | 2.50E-05 |
| GO:0000302 | response to reactive oxygen species | 23 | 5.60E-05 |
| GO:0009642 | response to light intensity | 23 | 0.00025 |
| GO:0006259 | DNA metabolic process | 56 | 0.0025 |
| GO:0010038 | response to metal ion | 61 | 0.0069 |
| GO:0009415 | response to water | 36 | 0.0094 |
| GO:0046686 | response to cadmium ion | 51 | 0.021 |
| GO:0006979 | response to oxidative stress | 45 | 0.037 |
| GO:0009414 | response to water deprivation | 33 | 0.037 |
| GO:0006260 | DNA replication | 25 | 0.043 |
